# Symptoms and risk factors for long COVID in non-hospitalized adults

**DOI:** 10.1038/s41591-022-01909-w

**Published:** 2022-07-25

**Authors:** Anuradhaa Subramanian, Krishnarajah Nirantharakumar, Sarah Hughes, Puja Myles, Tim Williams, Krishna M. Gokhale, Tom Taverner, Joht Singh Chandan, Kirsty Brown, Nikita Simms-Williams, Anoop D. Shah, Megha Singh, Farah Kidy, Kelvin Okoth, Richard Hotham, Nasir Bashir, Neil Cockburn, Siang Ing Lee, Grace M. Turner, Georgios V. Gkoutos, Olalekan Lee Aiyegbusi, Christel McMullan, Alastair K. Denniston, Elizabeth Sapey, Janet M. Lord, David C. Wraith, Edward Leggett, Clare Iles, Tom Marshall, Malcolm J. Price, Steven Marwaha, Elin Haf Davies, Louise J. Jackson, Karen L. Matthews, Jenny Camaradou, Melanie Calvert, Shamil Haroon

**Affiliations:** 1 Institute of Applied Health Research, University of Birmingham, Birmingham, UK; 2 Midlands Health Data Research UK, Birmingham, UK; 3 DEMAND Hub, University of Birmingham, Birmingham, UK; 4 Centre for Patient-Reported Outcomes Research, Institute of Applied Health Research, University of Birmingham, Birmingham, UK; 5 National Institute for Health and Care Research (NIHR) Applied Research Collaboration (ARC) – West Midlands, Birmingham, UK; 6 Birmingham Health Partners Centre for Regulatory Science and Innovation, University of Birmingham, Birmingham, UK; 7 NIHR Birmingham-Oxford Blood and Transplant Research Unit (BTRU) in Precision Transplant and Cellular Therapeutics, University of Birmingham, Birmingham, UK; 8 Clinical Practice Research Datalink, Medicines and Healthcare products Regulatory Agency, London, UK; 9 School of Sport, Exercise and Rehabilitation Sciences, University of Birmingham, Birmingham, UK; 10 Institute of Health Informatics, Faculty of Population Health Sciences, University College London, London, UK; 11 Warwick Medical School, University of Warwick, Coventry, UK; 12 School of Oral and Dental Sciences, University of Bristol, Bristol, UK; 13 NIHR Surgical Reconstruction and Microbiology Research Centre, University Hospital Birmingham and University of Birmingham, Birmingham, UK; 14 Institute of Cancer and Genomic Sciences, University of Birmingham, Birmingham, UK; 15 NIHR Birmingham Biomedical Research Centre, University Hospital Birmingham and University of Birmingham, Birmingham, UK; 16 University Hospitals Birmingham NHS Foundation Trust, Birmingham, UK; 17 Centre for Trauma Science Research, University of Birmingham, Birmingham, UK; 18 MRC-Versus Arthritis Centre for Musculoskeletal Ageing Research, Institute of Inflammation and Ageing, University of Birmingham, Birmingham, UK; 19 PIONEER HDR-UK Data Hub in acute care, University of Birmingham, Birmingham, UK; 20 UK SPINE, University of Birmingham, Birmingham, UK; 21 Institute of Immunology and Immunotherapy, University of Birmingham, Birmingham, UK; 22 Institute for Mental Health, University of Birmingham, Birmingham, UK; 23 Birmingham and Solihull Mental Health NHS Foundation Trust, Birmingham, UK; 24 Aparito Ltd, Wrexham, UK; 25 Patient and public involvement member, Birmingham, UK

**Keywords:** Respiratory signs and symptoms, Risk factors

## Abstract

Severe acute respiratory syndrome coronavirus-2 (SARS-CoV-2) infection is associated with a range of persistent symptoms impacting everyday functioning, known as post-COVID-19 condition or long COVID. We undertook a retrospective matched cohort study using a UK-based primary care database, Clinical Practice Research Datalink Aurum, to determine symptoms that are associated with confirmed SARS-CoV-2 infection beyond 12 weeks in non-hospitalized adults and the risk factors associated with developing persistent symptoms. We selected 486,149 adults with confirmed SARS-CoV-2 infection and 1,944,580 propensity score-matched adults with no recorded evidence of SARS-CoV-2 infection. Outcomes included 115 individual symptoms, as well as long COVID, defined as a composite outcome of 33 symptoms by the World Health Organization clinical case definition. Cox proportional hazards models were used to estimate adjusted hazard ratios (aHRs) for the outcomes. A total of 62 symptoms were significantly associated with SARS-CoV-2 infection after 12 weeks. The largest aHRs were for anosmia (aHR 6.49, 95% CI 5.02–8.39), hair loss (3.99, 3.63–4.39), sneezing (2.77, 1.40–5.50), ejaculation difficulty (2.63, 1.61–4.28) and reduced libido (2.36, 1.61–3.47). Among the cohort of patients infected with SARS-CoV-2, risk factors for long COVID included female sex, belonging to an ethnic minority, socioeconomic deprivation, smoking, obesity and a wide range of comorbidities. The risk of developing long COVID was also found to be increased along a gradient of decreasing age. SARS-CoV-2 infection is associated with a plethora of symptoms that are associated with a range of sociodemographic and clinical risk factors.

## Abstract (web summary)

A retrospective analysis of primary care records in the United Kingdom reveals individual symptoms associated with SARS-CoV-2 infections, which persisted for 12 weeks or more after infection, as well as risk factors associated with developing long COVID.

## Main

Infection with SARS-CoV-2 causes an acute multisystem illness referred to as COVID-19 ^1^. It is recognized that approximately 10% of individuals with COVID-19 develop persistent and often relapsing and remitting symptoms beyond 4 to 12 weeks after infection^2^. The presence of persistent symptoms in a previously infected individual is commonly referred to by several terms including post-COVID-19 condition, post-acute COVID-19 syndrome, post-acute sequelae of COVID-19 (PASC) and long COVID^3–5^. The UK National Institute for Health and Care Excellence (NICE) makes a distinction between disease occurring from 4 to 12 weeks after infection (ongoing symptomatic COVID-19) and symptoms persisting beyond 12 weeks (post-acute COVID-19 syndrome)^4^. The World Health Organization (WHO) defines it as a condition characterized by symptoms impacting everyday life, such as fatigue, shortness of breath and cognitive dysfunction, which occur after a history of probable or confirmed SARS-CoV-2 infection^6^. Symptoms usually occur 3 months from the onset of acute COVID-19 symptoms, last for at least 2 months and cannot be explained by an alternative diagnosis.

Long COVID has been associated with a broad range of symptoms and health impacts^5,7–9^. A previous study showed that symptoms of long COVID, although commonly observed among patients with other viral infections such as influenza, occur more frequently following infection with SARS-CoV-2^10^. Several systematic reviews have shown the most prevalent symptoms to be fatigue, shortness of breath, muscle pain, joint pain, headache, cough, chest pain, altered smell, altered taste and diarrhea^9,11–13^; however, previous studies were often based on self-reported symptoms or lacked a control group, making it difficult to make inferences about whether the reported symptoms were due to SARS-CoV-2 infection, pre-existing comorbidities or societal effects related to the pandemic. Furthermore, many previous studies were conducted in hospitalized cohorts^14,15^, and population-level data on the potential breadth of symptoms experienced by non-hospitalized individuals with SARS-CoV-2 infection are scarce. Large-scale studies leveraging routinely available healthcare data with closely matched control populations are needed to elucidate which symptoms are independently associated with the long-term effects of COVID-19.

There is also a need to gain a better understanding of the risk factors that contribute toward the development of long COVID, which was highlighted as a research priority on the recently updated NICE guideline on managing the long-term effects of COVID-19^4^. Previous studies suggested that higher risk of developing long COVID was observed with a gradient increase in age, female sex, hospital admission during acute COVID-19 (including the need for oxygen therapy), symptom load (including dyspnea at presentation and chest pain), abnormal auscultation findings and the presence of comorbidities such as asthma^16–19^. Large-scale population-based studies with appropriate control groups are required to assess the long-term symptoms that are specifically attributable to SARS-CoV-2 infection and their association with a wide range of demographic and clinical risk factors in non-hospitalized individuals. Such studies are needed to understand the breadth of symptoms that contribute to long COVID to inform clinical management and help healthcare providers identify population groups at higher risk of reporting persistent symptoms.

Here, we did a large-scale analysis of primary care data from the UK to investigate a comprehensive range of symptoms previously reported to be associated with long COVID by epidemiological studies, patients and clinicians. We aimed to assess their association with confirmed SARS-CoV-2 infection at least 12 weeks after infection in non-hospitalized adults, compared to a propensity score-matched cohort of patients with no recorded evidence of SARS-CoV-2 infection. We also assessed associations between demographic and clinical risk factors, including comorbidities, with the development of long COVID and characterized dominant symptom clusters.

## Results

### Participants

**Extended Data Fig. 1:**
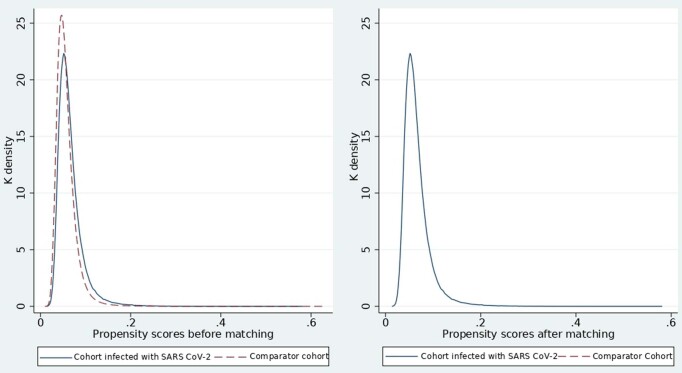
Kernel density plot of propensity scores of patients infected with SARS CoV-2 and comparator cohort of patients with no recorded evidence of SARS CoV-2 infection, before and after propensity score matching.

A total of 486,149 non-hospitalized individuals had a coded record of SARS-CoV-2 infection, and 8,030,224 had no records of either suspected or confirmed COVID-19 during the study period between 31 January 2020 and 15 April 2021. From the pool of patients with no recorded evidence of SARS-CoV-2 infection, 1,944,580 individuals were propensity score-matched to patients infected with SARS-CoV-2. Kernel density plots of the propensity scores of both the cohorts, before and after matching, are presented in Extended Data Fig. 1. The total follow-up time was 0.29 years (interquartile range (IQR) 0.24–0.42) for the cohort of patients infected with SARS-CoV-2 and 0.29 (IQR 0.24–0.41) for the cohort of patients with no recorded evidence of SARS-CoV-2 infection.

The cohorts of patients were well matched in terms of sociodemographic characteristics, smoking status, body mass index (BMI), comorbidities and baseline recording of symptoms, indicated by standardized mean difference (SMD) < 0.1 for all variables (Table 1 and Supplementary Table 1). The mean age was 43.8 years (s.d. 16.9), and 55.3% of participants were female. Of the participants, 64.7% were white, 12.2% were Asian origin from India, Pakistan, China, Cambodia, Thailand, Vietnam, Malaysia, Sri Lanka, Nepal, Bangladesh, Japan or Taiwan, 4.0% were Black Afro-Caribbean and 16.2% had missing ethnicity data. Overall, 53.8% were overweight or obese (with BMI data missing for 13.0%), and 22.5% were current smokers (with smoking data missing for 4.3%).

**Table 1:** Baseline characteristics of patients infected with SARS-CoV-2 and propensity-matched comparator cohort of patients with no recorded evidence of SARS-CoV-2 infection

	Cohort of patients infected with SARS-CoV-2 (*n* = 486,149)	Comparator cohort (*n* = 1,944,580)	Standardized differences
**Mean age at index date (s.d.)**	44.1 (17.0)	43.8 (16.9)	0.015
**Sex,** ***n*** **(%)**			
Female	268,367 (55.2)	1,075,963 (55.3)	0.003
Male	217,782 (44.8)	868,617 (44. 7)	
**Ethnic group,** ***n*** **(%)**			
White	313,561 (64.5)	1,258,392 (64.7)	0.004
Asian^a^	59,477 (12.2)	237,133 (12.2)	
Black Afro-Caribbean	19,835 (4.1)	78,501 (4.0)	
Mixed ethnicity	7,357 (1.5)	29,614 (1.5)	
Other^b^	6,896 (1.4)	26,966 (1.4)	
Missing	79,023 (16.3)	313,974 (16.2)	
**Socioeconomic status IMD quintile,** ***n*** **(%)**			0.003
1 (least deprived)	82,538 (17.0)	331,229 (17.0)	
2	86,164 (17.7)	346,054 (17.8)	
3	89,470 (18.4)	358,650 (18.4)	
4	106,578 (21.9)	426,153 (21.9)	
5 (most deprived)	112,656 (23.2)	448,126 (23.0)	
Missing	8,743 (1.8)	34,368 (1.8)	
**BMI (kg** **m**^**−2**^**),** ***n*** **(%)**			
<18.5	13,261 (2.7)	52,322 (2.7)	0.001
18.5–25	148,295 (30.5)	590,747 (30.4)	
25–30	138,771 (28.5)	558,287 (28.7)	
>30	121,943 (25.1)	489,389 (25.2)	
Missing	63,879 (13.1)	253,835 (13.1)	
**Smoking status**			
Never smoked	177,064 (36.4)	714,045 (36.7)	0.009
Ex-smoker	176,899 (36.4)	710,255 (36.5)	
Current smoker	110,848 (22.8)	436,212 (22.4)	
Missing	21,338 (4.4)	84,068 (4.3)	
**Comorbidities**			
Depression	107,392 (22.1)	428,797 (22.1)	*0.001*
Anxiety	98,849 (20.3)	395,365 (20.3)	0.000
Asthma	97,509 (20.1)	390,401 (20.1)	0.000
Eczema	94,313 (19.4)	378,604 (19.5)	0.002
Hay fever	87,691 (18.0)	352,090 (18.1)	0.002
Hypertension	73,901 (15.2)	291,389 (15.0)	0.006
Migraine	53,881 (11.1)	215,733 (11.1)	0.000
Osteoarthritis	53,694 (11.0)	211,062 (10.9)	0.006
Fragility fracture	46,608 (9.6)	186,194 (9.6)	0.000
Arrhythmias	34,811 (7.2)	136,280 (7.0)	0.006
**Calendar year of index date,** ***n*** **(%)**			
2020	275,169 (56.6)	1,077,126 (55.4)	0.024
2021	210,980 (43.4)	867,454 (44.6)	
**COVID-19 vaccine status at index date,** ***n*** **(%)**			
Vaccine dose 1	21,932 (4.5)	92,355 (4.7)	*0.013*
Vaccine dose 2	685 (0.1)	5,964 (0.3)	*0.035*
ChAdOx1-S	8,210 (1.7)	32,183 (1.7)	0.003
BNT162b2	12,792 (2.6)	56,559 (2.9)	0.017
CX-024414	0 (0)	3 (0)	0.002

Socioeconomic status measured using the Index of Multiple Deprivation (IMD); standardized difference of less than 0.1 indicates a relatively small imbalance. Cohort of patients with SARS-CoV-2 infection included participants with a positive PCR with reverse transcription (RT–PCR) or antigen test for SARS-CoV-2. The comparator cohort included participants with no records of either confirmed or suspected COVID-19.

^a^The Asian category consisted of participants with origin from all over Asia, including India, Pakistan, China, Cambodia, Thailand, Vietnam, Malaysia, Sri Lanka, Nepal, Bangladesh, Japan or Taiwan. ^b^The ‘other’ ethnicity category consisted of patients with native American, Middle Eastern or Polynesian origin.

The most common comorbidities were depression (22.1%), anxiety (20.3%), asthma (20.1%), eczema (19.5%) and hay fever (18.1%). A full list of comorbidities is provided in Supplementary Table 1. Overall, 56.6% of the patients infected with SARS-CoV-2 had been diagnosed in 2020 and 43.4% in 2021. 4.5% of the patients infected with SARS-CoV-2 and 4.7% of the patients with no recorded evidence of SARS-CoV-2 infection had received at least a single dose of a COVID-19 vaccine before the index date. The most common vaccine before the index date was the BNT162b2 (BioNTech-Pfizer; 2.8%) followed by ChAdOx1 nCoV-19 (Oxford-AstraZeneca; 1.7%).

### Symptoms

In the 3–12-month period before the index date, the reporting of symptoms between the patients infected with SARS-CoV-2 and propensity score-matched cohort of patients with no recorded evidence of SARS-CoV-2 infection were similar. Of the 115 symptoms, statistically significant differences between the two groups at baseline were observed only for bowel incontinence and sore throat, after adjustment for age, sex, ethnic group, socioeconomic status, smoking status and BMI using logistic regression (Supplementary Table 2).

**Fig. 1:**
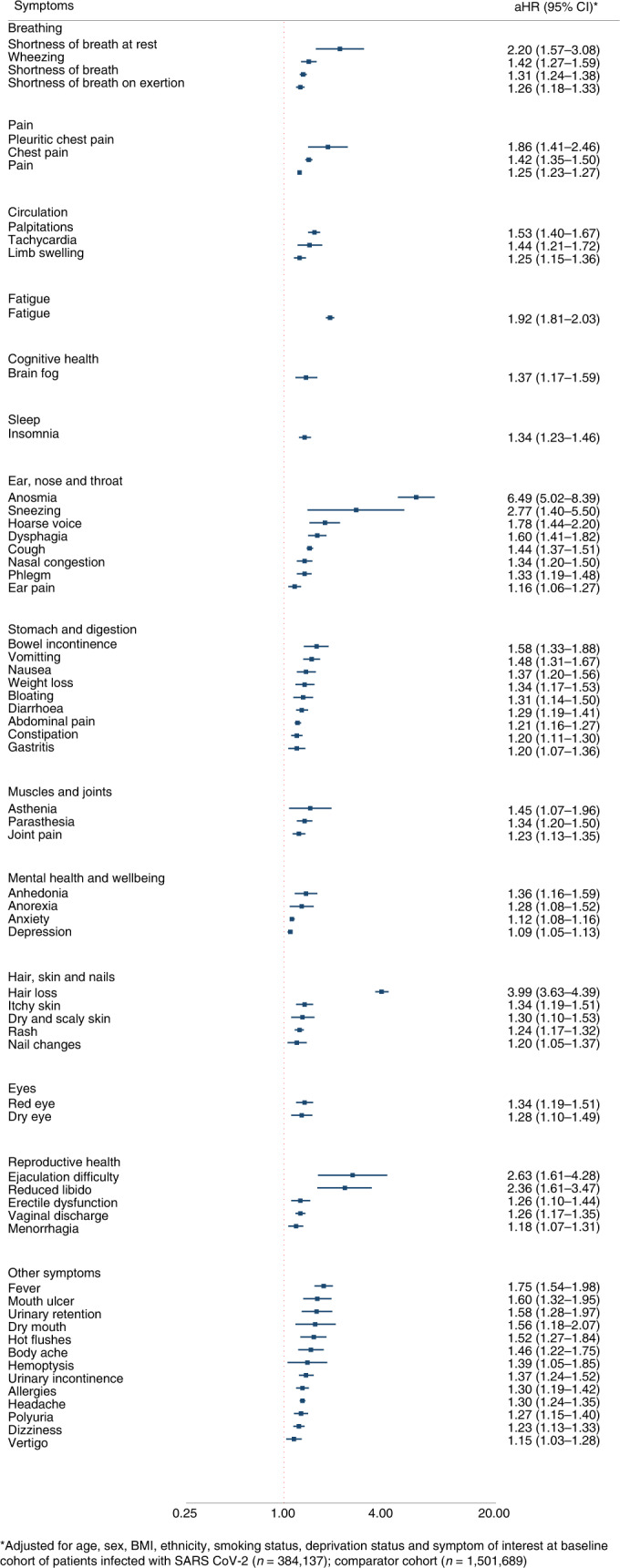
Symptoms associated with SARS-CoV-2 ≥ 12 weeks after infection.

At 12 weeks after the index date, a history of SARS-CoV-2 infection was significantly associated with a total of 62 symptoms, after adjustment for age, sex, ethnic group, socioeconomic status, smoking, BMI and baseline symptoms (Supplementary Table 3a). These 62 symptoms spanned 14 of the 15 domains considered (Fig. 1). Of the patients with a minimum of 12 weeks of follow-up, 20,864 out of 384,137 (5.4%) patients infected with SARS-CoV-2 and 65,293 out of 1,501,689 (4.3%) patients with no recorded evidence of SARS-CoV-2 infection reported at least one of the symptoms included in the WHO case definition for long COVID (aHR 1.26, 95% CI 1.25–1.28) (Supplementary Table 3a). Patients infected with SARS-CoV-2 were more likely to report more than one symptom after 12 weeks from the index date compared to patients with no recorded evidence of SARS-CoV-2 infection (one symptom (5.6% versus 4.7%), two symptoms (3.6% versus 2.9%) and three or more symptoms (4.9% versus 4.0%)) (Supplementary Table 3b).

The symptoms with the largest aHRs were anosmia (aHR 6.49, 95% CI 5.02–8.39), hair loss (3.99, 3.63–4.39), sneezing (2.77, 1.40–5.50), ejaculation difficulty (2.63, 1.61–4.28), reduced libido (2.36, 1.61–3.47), shortness of breath at rest (2.20, 1.57–3.08), fatigue (1.92, 1.81–2.03), pleuritic chest pain (1.86, 1.41–2.46), hoarse voice (1.78, 1.44–2.20) and fever (1.75, 1.54–1.98).

The association of SARS-CoV-2 infection with these 62 significantly associated symptoms was even larger at 0–4 weeks and 4–12 weeks, and the size of the aHRs reduced with increasing time from the index date. A full list of the aHRs for all 115 symptoms included in the analysis at 0–4 weeks, 4–12 weeks and beyond 12 weeks is presented in Supplementary Tables 3a, 4 and 5.

**Extended Data Fig. 2:**
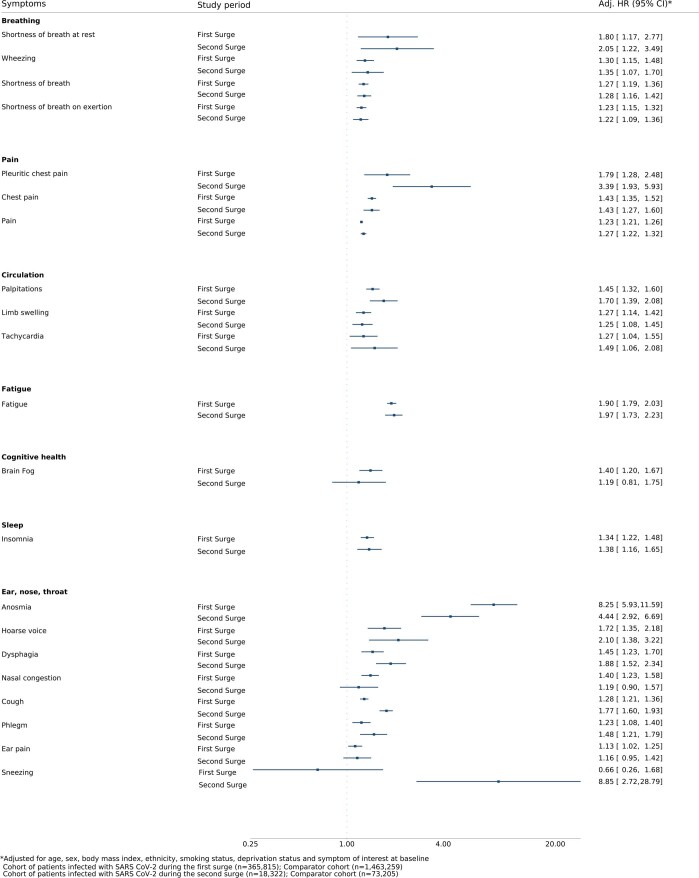
Symptoms associated with SARS CoV-2 ≥ 12 weeks post-infection before and after 31 August 2020 (first and second surges of the pandemic in the UK) – Part A.

**Extended Data Fig. 3:**
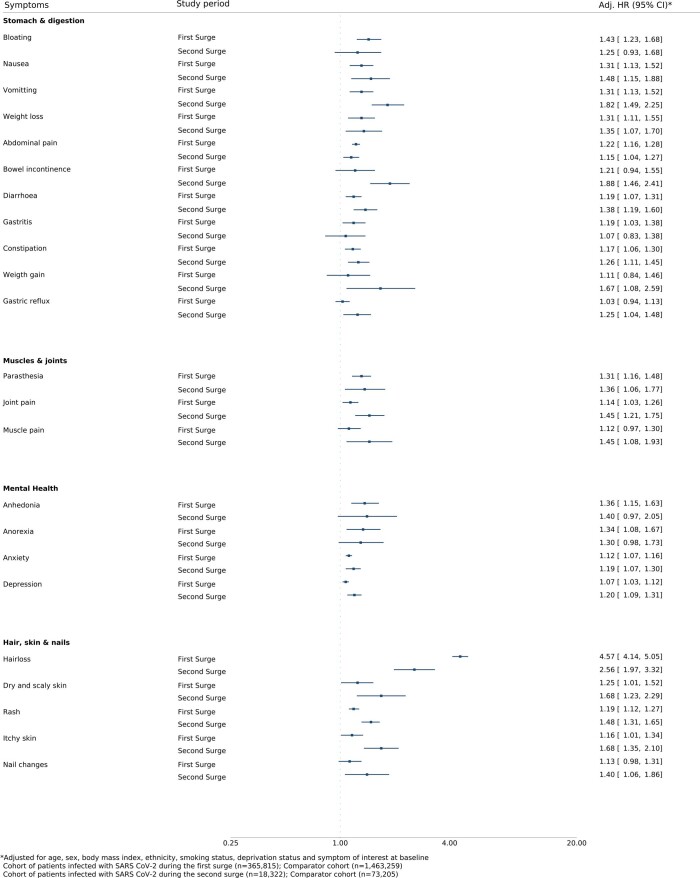
Symptoms associated with SARS CoV-2 ≥ 12 weeks post-infection before and after 31 August 2020 (first and second surges of the pandemic in the UK) – Part B.

**Extended Data Fig. 4:**
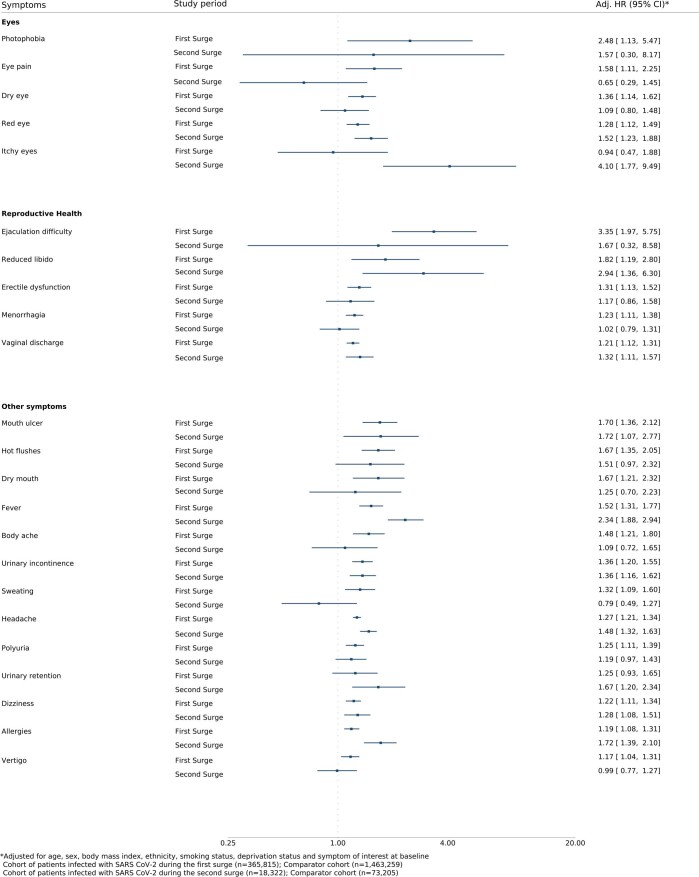
Symptoms associated with SARS CoV-2 ≥ 12 weeks post-infection before and after 31 August 2020 (first and second surges of the pandemic in the UK) – Part C.

In the post hoc-subgroup analysis of patients infected during the first and second surges of the pandemic in the UK (31 January 2020 to 31 August 2020 and 1 September 2020 to 15 April 2021, when the dominant variant of concern was B.1.1.7) and their propensity score-matched patients, the association between SARS-CoV-2 infection and the reported symptoms is more pronounced among those infected during the second wave of the pandemic. For example, SARS-CoV-2 infection during the first surge of the pandemic was associated with only a 28% relative increase in the reporting of cough after 12 weeks from the index date compared to propensity score-matched patients (aHR 1.28, 95% CI 1.21–1.36), whereas infection during the second wave was associated with a 77% relative increase in the reporting of cough compared to corresponding propensity score-matched patients (aHR 1.77, 95% CI 1.60–1.93). Similar trends were also observed for sneezing, rash, itchy skin, fever and allergies (Extended Data Figs. 2–4).

### Risk factors for long COVID symptoms

The risk factor analysis included 384,137 individuals infected with SARS-CoV-2 with a minimum of 12 weeks of follow-up. When using the WHO definition of long COVID, several sociodemographic and clinical risk factors were significantly associated with the incidence of long COVID (Table 2 and Supplementary Table 6). Women were at increased risk compared to men (aHR 1.52, 95% CI 1.48–1.56). Older age above 30 years was associated with a higher risk of reporting long COVID symptoms in the univariate analysis; however, after adjusting for baseline covariates, older age was associated with a lower risk, with those aged 30–39 years having a 6% lower risk (0.94, 0.90–0.97) and those aged ≥70 years having a 25% lower risk (0.75, 0.70–0.81) compared to those aged 18–30 years.

**Table 2:** Risk factors associated with the development of long COVID (WHO definition)

Risk factor	Total numbers per strata (*n* = 384,137)	Long COVID symptoms (*n* = 29,869) (7.78) *n* (%)	Unadjusted HR (95% CI)	Adjusted HR^a^ (95% CI)
**Sex**				
Men	171,593	9,090 (5.3)	Ref.	Ref.
Women	212,544	20,779 (9. 8)	1.86 (1.81–1.90)	1.52 (1.48–1.56)
**Age (years)**				
***18–29***	95,969	6,932 (7.2)	Ref.	Ref.
***30–39***	78,302	5,805 (7.4)	1.13 (1.10–1.18)	0.94 (0.90–0.97)
***40–49***	75,349	5,784 (7.7)	1.14 (1.10–1.18)	0.89 (0.86-0.93)
***50–59***	73,262	5,485 (7.5)	1.07 (1.04–1.11)	0.80 (0.77–0.83)
***60–69***	35,932	2,790 (7.8)	1.09 (1.05–1.14)	0.74 (0.70–0.78)
***≥70***	25,323	3,073 (12.1)	1.39 (1.33–1.45)	0.75 (0.70–0.81)
**Ethnicity**				
***White***	246,717	20,462 (8.3)	Ref.	Ref.
***Asian***^***b***^	47,788	3,647 (7.6)	0.90 (0.82–0.99)	0.99 (0.89–1.09)
***Black***	15,846	1,053 (6.7)	1.01 (0.91–1.11)	1.21 (1.10–1.34)
***Mixed***	5,976	407 (6.8)	0.98 (0.92–1.04)	1.14 (1.07–1.22)
***Other***^***c***^	5,438	404 (7.4)	0.94 (0.91–0.97)	1.06 (1.03–1.10)
***Missing***	62,372	3,896 (6.3)	0.74 (0.71–0.76)	0.92 (0.88–0.95)
**BMI (kg** **m**^**−2**^**)**				
***<18.5***	10,312	762 (7.4)	0.93 (0.86–1.00)	0.93 (0.86–1.00)
***18.5–25***	117,630	8,849 (7.5)	Ref.	Ref.
***25–30***	109,707	8,612 (7.9)	1.06 (1.03–1.09)	1.07 (1.04–1.10)
***>30***	95,799	9,233 (9.6)	1.29 (1.25–1.33)	1.10 (1.07–1.14)
***Missing***	50,689	2,413 (4.8)	0.63 (0.60–0.65)	0.91 (0.86–0.95)
**Smoking status**				
***Non-smoker***	141,967	9,671 (6.8)	Ref.	Ref.
***Ex-smoker***	139,294	12,407 (8.9)	1.33 (1.29–1.36)	1.08 (1.05–1.11)
***Current smoker***	85,765	7,072 (8.3)	1.31 (1.27–1.35)	1.12 (1.08–1.15)
***Missing***	17,111	719 (4.2)	0.61 (0.56–0.65)	0.90 (0.83–0.97)
**Socioeconomic status quintile (IMD)**				
***1 (least deprived)***	66,564	4,392 (6.6)	Ref.	Ref.
***2***	68,657	4,963 (7.2)	1.09 (1.05–1.13)	1.05 (1.00–1.09)
***3***	70,699	5,486 (7.8)	1.19 (1.14–1.24)	1.10 (1.05–1.14)
***4***	84,002	6,523 (7.8)	1.20 (1.16–1.25)	1.07 (1.03–1.11)
***5 (most deprived)***	87,270	7,883 (9.0)	1.33 (1.28–1.38)	1.11 (1.07–1.16)
***Missing***	6,945	622 (9.0)	1.28 (1.7–1.39)	1.10 (1.01–1.20)
**Symptoms recorded before COVID-19**	78,880	13,207 (16.7)	2.92 (2.85–2.99)	2.07 (2.02–2.12)
**Comorbidities**				
COPD	8,040	1,741 (21.7)	2.71 (2.58–2.85)	1.55 (1.47–1.64)
BPH	4,961	596 (12.0)	1.39 (1.28–1.51)	1.39 (1.28–1.52)
Fibromyalgia	4,031	900 (22.3)	3.17 (2.97–3.39)	1.37 (1.28–1.47)
Anxiety	77,753	10,481 (13.5)	2.17 (2.12–2.23)	1.35 (1.31–1.39)
Erectile dysfunction	16,678	1,551 (9.3)	1.15 (1.09–1.21)	1.33 (1.26–1.41)
Depression	83,903	11,222 (13.4)	2.22 (2.17–2.27)	1.31 (1.27–1.34)
Migraine	43,043	5,597 (13.0)	1.88 (1.83–1.94)	1.26 (1.22–1.30)
Multiple sclerosis	791	98 (12.4)	1.52 (1.25–1.85)	1.26 (1.03–1.53)
Celiac disease	1,669	207 (12.4)	1.58 (1.38–1.81)	1.25 (1.09–1.43)
Learning disability	3,283	295 (9.0)	1.22 (1.09–1.37)	1.24 (1.11–1.40)
IBS	27,492	3,691 (13.4)	1.84 (1.78–1.91)	1.20 (1.15–1.24)
Endometriosis	5,727	800 (14.0)	1.92 (1.79–2.06)	1.19 (1.11–1.28)
Low Hb	20,039	2,683 (13.4)	1.78 (1.71–1.85)	1.18 (1.13–1.23)
Deafness	3,767	514 (13.6)	1.53 (1.40–1.67)	1.16 (1.06–1.27)
Eating disorder	3,488	504 (14. 5)	1.92 (1.75–2.09)	1.16 (1.06–1.27)
Substance misuse	6,449	775 (12.0)	1.69 (1.58–1.82)	1.15 (1.07–1.23)
Back pain	5,483	718 (13.1)	1.76 (1.64–1.90)	1.15 (1.07–1.24)
Asthma	76,946	8,527 (11.1)	1.59 (1.55–1.63)	1.15 (1.12–1.18)
Chronic sinusitis	6,838	873 (12.8)	1.63 (1.52–1.74)	1.14 (1.07–1.22)
PCOS	9,599	1,166 (12.2)	1.73 (1.63–1.84)	1.14 (1.07–1.21)

^a^aHRs estimated using a multivariable Cox proportional hazards model, including age, sex, ethnic group, socioeconomic status, index year, vaccination status, symptoms recorded before COVID-19 and comorbidities. ^b^The Asian category consisted of participants with origin from all over Asia including India, Pakistan, China, Cambodia, Thailand, Vietnam, Malaysia, Sri Lanka, Nepal, Bangladesh, Japan or Taiwan. ^c^The other ethnicity category consisted of patients with native American, Middle Eastern or Polynesian origin.

COPD, chronic obstructive pulmonary disease; BPH, benign prostatic hyperplasia; IBS, irritable bowel syndrome; Hb, hemoglobin; PCOS; polycystic ovary syndrome; Ref., reference.

There were associations between the risk of reporting long COVID symptoms and certain ethnic minority groups in the multivariable model, with increased risks seen in Black Afro-Caribbean ethnic groups (aHR 1.21, 95% CI 1.10–1.34), mixed ethnicity (1.14, 1.07–1.22) and other minority ethnic groups comprising of patients with native American, Middle Eastern or Polynesian origin (1.06, 1.03–1.10), as compared to white ethnic groups. The risk also increased with increasing levels of socioeconomic deprivation, with a 11% increased risk (1.11, 1.07–1.16) in those who were most socioeconomically deprived compared to those least deprived.

Smokers and former smokers were at increased risk of reporting long COVID symptoms (aHR 1.12, 95% CI 1.08–1.15 and 1.08, 1.05–1.11, respectively), compared to those who had never smoked. Baseline BMI in the overweight or obese range was also associated with an increased risk of persistent symptoms, with those who had a BMI of greater than 30 kg m^−2^ having a 10% relative increase in risk of reporting long COVID symptoms compared to those with a BMI of 18.5–25 kg m^−2^ (aHR 1.10, 1.07–1.14).

A wide range of comorbidities at baseline were also associated with an increased risk of long COVID symptoms. The comorbidities with the largest associations were COPD (aHR 1.55, 95% CI 1.47–1.64), benign prostatic hyperplasia (1.39, 1.28–1.52), fibromyalgia (1.37, 1.28–1.47), anxiety (1.35, 1.31–1.39), erectile dysfunction (1.33, 1.26–1.41), depression (1.31, 1.27–1.34), migraine (1.26, 1.22–1.30), multiple sclerosis (1.26, 1.03–1.53), celiac disease (1.25, 1.09–1.43) and learning disability (1.24, 1.11–1.40). A full list of the aHRs for the included comorbidities is provided in Supplementary Table 6.

When using our alternative definition of long COVID that consisted of having at least one of the symptoms that were statistically associated with a history of SARS-CoV-2 infection ≥12 weeks after infection, the risk factor patterns were largely still observed (Supplementary Table 7). Females, ethnic minority groups, increasing socioeconomic deprivation, smoking and former smoking, high BMI and a wide range of comorbidities were all associated with an increased risk of reporting symptoms ≥12 weeks after infection. Risk of reporting symptoms was also found to be increased along a gradient of decreasing age.

### Symptom clusters among patients with long COVID

**Extended Data Fig. 5:**
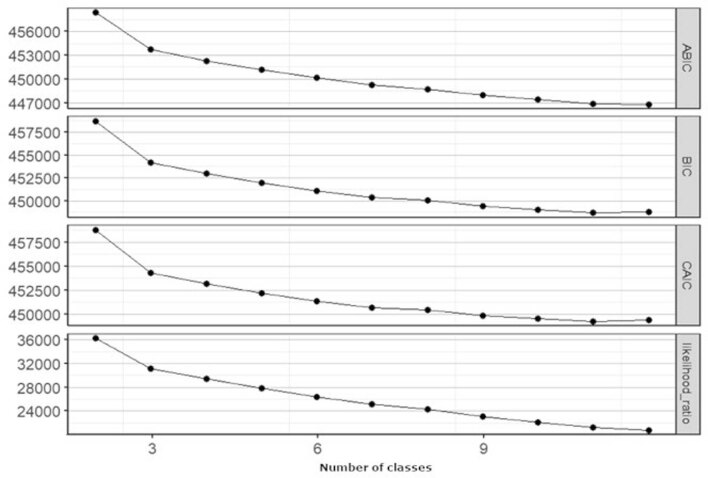
Elbow plot to determine the optimal number of classes.

**Extended Data Fig. 6:**
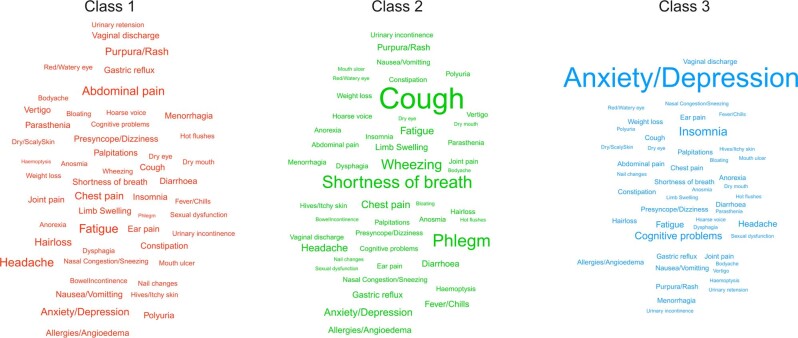
Symptom clusters among patients with long COVID from latent class analysis.

A three-class model achieved the optimal fit in a latent class analysis of 50 consolidated symptoms (Supplementary Table 8) among patients with SARS-CoV-2 infection who reported at least one of the 62 symptoms associated with COVID-19 beyond 12 weeks after infection (*n* = 50,832; Extended Data Fig. 5). Latent class proportions and the probabilities of symptoms conditional to class membership (*ρ*) are given in Supplementary Table 9a,b. A word cloud of symptom names was generated for the three classes, where the text size of the symptoms is directly proportional to the *ρ* parameter (Extended Data Fig. 6). Among patients with SARS-CoV-2 infection with persistent symptoms, 80.0% belonged to class 1 (dominated by a broad spectrum of symptoms including pain, fatigue and rash), 5.8% to class 2 (dominated by cough, shortness of breath and phlegm) and 14.2% to class 3 (dominated by depression, anxiety, insomnia and brain fog).

The baseline characteristics of patients within each of the latent classes is presented in Supplementary Table 10. A multinomial logistic regression model was performed for the polytomous class membership outcome among those with SARS-CoV-2 infection (Supplementary Table 11). Patients from all the classes were more likely to be socioeconomically deprived and to be women compared to patients without persistent symptoms. Compared to patients without persistent symptoms, members of latent class 3 (dominated by anxiety, depression, insomnia and brain fog) were more likely to be younger, whereas members of the other latent classes were more likely to be older compared to patients without persistent symptoms. Members of latent class 2 and 3 were more likely to be white, whereas members of latent class 1 (dominated by a broad spectrum of symptoms including pain, fatigue and rash) were more likely to be of Asian origin or from other ethnic minority groups.

## Discussion

Individuals with confirmed SARS-CoV-2 infection were at increased risk of reporting a wide range of symptoms at ≥12 weeks after infection, compared to propensity score-matched patients with no record of suspected or confirmed SARS-CoV-2 infection, after accounting for both sociodemographic and clinical characteristics and the reporting of symptoms before infection. The symptoms most associated with SARS-CoV-2 infection included some that are already recognized in previous studies^12^, such as anosmia, shortness of breath, chest pain and fever, but also included a range of other symptoms that have previously not been widely reported such as hair loss and sexual dysfunction. Previous SARS-CoV-2 infection was independently associated with the reporting to primary care of 20 of the 33 symptoms included in the WHO case definition and an additional 42 symptoms, beyond 12 weeks from infection. SARS-CoV-2 infection was associated with a 26% relative increase in risk of reporting at least one of the symptoms included in the WHO case definition for long COVID.

Among those with a history of confirmed SARS-CoV-2 infection, several risk factors were associated with reporting symptoms 12 weeks or more after infection. Female sex, a gradient of decreasing age, belonging to a Black, mixed ethnicity or other ethnic minority group, socioeconomic deprivation, smoking, high BMI and the presence of a wide range of comorbidities were associated with increased risk of both symptoms included in the WHO definition of long COVID and symptoms statistically associated with SARS-CoV-2 infection reported 12 weeks or more after infection.

Among those with a confirmed SARS-CoV-2 infection and who reported at least one symptom that was statistically associated with SARS-CoV-2 infection at least 12 weeks after infection, three major clusters of phenotypes of long COVID were observed. These included patients with symptoms dominated by (1) a broad spectrum of symptoms, including pain, fatigue and rash (80.0%); (2) respiratory symptoms, including cough, shortness of breath and phlegm (5.8%); and (3) mental health and cognitive symptoms, including anxiety, depression, insomnia and brain fog (14.2%).

A key strength of the study is the large sample size, which included 486,149 adults with a confirmed diagnosis of SARS-CoV-2 infection and 1.9 million propensity score-matched patients with no recorded evidence of SARS-CoV-2 infection. The large sample size provided adequate statistical power to assess differences in the reporting of a wide range of symptoms between the two cohorts and estimation of the association between reporting of symptoms and important sociodemographic and clinical risk factors with a high level of precision. Another key strength of the study is the inclusion of a comparator group that did not have either suspected or confirmed SARS-CoV-2 infection and had been propensity score-matched for sociodemographic factors, previously reported symptoms and over 80 comorbidities. This enabled us to assess the independent association between exposure to SARS-CoV-2 and the reporting of symptoms ≥12 weeks after infection, after accounting for many important confounders. A further strength is the large number of symptoms included in the analysis, which was based on a previous systematic review of the literature^11^, a scoping review of long COVID questionnaires and an extensive consultation with patients and clinicians^20﻿]^. Symptom code lists were developed rigorously with systematic searches for relevant SNOMED CT codes with extensive clinical input. We also assessed the outcome of long COVID using the WHO case definition as well as a new definition that incorporated symptoms that were statistically associated with a history of SARS-CoV-2 infection.

A key limitation of the study is the use of routinely coded healthcare data. Coded symptom data in primary care records is likely to underrepresent the true symptom burden experienced by individuals with long COVID. This could be due to reduced access to primary care (especially during the first surge of the pandemic), patients not consulting their general practitioner (GP) about symptoms or the reason for the GP consultation being unrelated to COVID-19, thereby leading patients to underreport the full extent and breadth of their symptoms. In addition, much of a patient’s clinical history, in terms of the symptoms reported, are recorded as free text, rather than as SNOMED CT codes^21^. The symptom data we used for the study thus cannot be used to make inferences about the absolute prevalence of these symptoms; however, as this underrepresentation would be expected to affect both the infected and propensity score-matched comparator cohorts equally, the data used in the present analysis can still be used to examine relative differences in the reporting of symptoms between patients infected with SARS-CoV-2 and patients with no recorded evidence of SARS-CoV-2 infection. Conversely, with the evolving awareness of long COVID, it is possible that patients with a history of COVID-19 may have been more likely than those without to access primary care and alert clinicians of their symptoms, which could potentially lead to an inflation of the observed effect sizes. This is potentially supported by the increased aHRs observed for symptoms such as cough, sneezing, fever and allergies among patients who were infected during the second surge of the pandemic, compared to those infected during the first surge, although this could also potentially be attributed to other reasons, such as changes in the dominant variants.

Another limitation of the study is potential misclassification bias. Community testing for SARS-CoV-2 was very limited during the first surge of the pandemic, and many hospitalized individuals who were not hospitalized with COVID-19 were not tested. Furthermore, antigen test positive results may not be routinely coded within primary care. There is some evidence that as much as 20–30% of SARS-CoV-2 test positive cases may be missing from primary care records^22,23^. It is therefore possible that some members of our propensity score-matched comparator cohort had been infected with SARS-CoV-2 but had simply not been tested or coded as confirmed COVID-19 within primary care. We attempted to account for this bias by excluding individuals from the comparator cohort if they had a coded diagnosis of suspected COVID-19; however, this is unlikely to be completely sensitive in identifying individuals with unverified SARS-CoV-2 infection from the comparator cohort, which would potentially have the effect of attenuating the observed effect sizes. Similarly, it is possible that some members of our cohort were hospitalized, as we were limited to using SNOMED CT codes for hospitalization within primary care records rather than using linked Hospital Episode Statistics data, of which timely access was unavailable for our study.

Finally, we were unable to incorporate all aspects of the WHO clinical case definition for long COVID, such as ‘impact on everyday functioning’ due to the lack of data on these domains within coded primary care data. Our findings support the results from our previous systematic review and meta-analysis on long COVID symptoms^11^. That review found the most prevalent symptoms to be fatigue, shortness of breath, muscle pain, joint pain, headache, cough, chest pain, altered sense of smell, altered taste and diarrhea. Our current analysis was not able to assess symptom prevalence but rather the relative difference in symptoms between a large sample of individuals with and without recorded evidence of SARS-CoV-2 infection at ≥12 weeks after infection. We similarly identified anosmia, shortness of breath, fatigue and chest pain to be symptoms significantly associated with SARS-CoV-2 infection. By contrast, we also identified new symptoms such as hair loss, sneezing, symptoms of sexual dysfunction (difficulties ejaculating and reduced libido), hoarse voice and fever as significantly associated. Also, like our review^11^, we found that female sex and the presence of a range of comorbidities were associated with an increased risk of developing persistent symptoms; however, it is likely that pre-existing comorbidities may have influenced the likelihood of GP consultations and symptom reporting.

In contrast to our review, the present analysis found that risk of reporting symptoms at ≥12 weeks after infection increased along a gradient of decreasing age in our cohort. This could partly be due to the adjustment for an extensive range of comorbidities or the differences in the populations studied. Most studies included in our review were based on hospitalized cohorts, whereas our present study excluded hospitalized patients. Older patients with COVID-19 were more likely to be hospitalized than younger patients and, therefore, to be excluded from our study. Older non-hospitalized patients might, therefore, have had mild disease with low symptom burden.

We also found that patients from Black, mixed ethnicity and other minority ethnic backgrounds were at increased risk of persistent symptoms. This contradicts the findings from the analysis of the COVID-19 Infection Survey data, which found a lower prevalence of long COVID among all ethnic minority subgroups compared to those of white ethnicity^24^; however, the COVID-19 Infection Survey analysis included children, was restricted to those living in private residences and considered self-reported diagnosis of long COVID, defined as unexplained persistence of symptoms, 4 weeks after SARS-CoV-2 infection.

An international online cohort study of people with confirmed and suspected long COVID found that respondents reported an average of 56 symptoms across an average of nine organ systems^8^. A Norwegian prospective study of 312 home-isolated patients found persistent symptoms 6 months after infection^25^. Both studies were comprehensive analyses of symptom burden but lacked a control group and were therefore unable to make strong inferences about the relative contribution of SARS-CoV-2 infection to these symptoms over and above pre-existing health conditions or psychosocial effects related to the pandemic; however, like these studies, we also found that individuals with a history of confirmed SARS-CoV-2 reported a broad range of symptoms, with a total of 62 symptoms being associated at 12 or more weeks after infection. We were also able to control for potential confounders, including whether the symptoms of interest were reported before infection.

The COVID Symptom Study provided data on self-reported symptoms among participants enrolled on an app^16^. Among those with symptoms persisting 28 d or longer after infection, key symptoms included fatigue, headache, dyspnea and anosmia, which were all also significantly associated at ≥12 weeks in our cohort. The COVID Symptom Study also found that long COVID was associated with increasing BMI and female sex, which is in keeping with our findings; however, the study also found that the risk of reporting long COVID symptoms increased with age, whereas our study observed the opposite trend after adjustment for a comprehensive range of potential confounders. Although the COVID Symptom Study is community-based, it includes individuals with a history of hospitalized and non-hospitalized COVID-19, so the reasons for the discrepant age trend may be due to the exclusion of older patients in our study who are more likely to be hospitalized.

One of the largest population-based surveys on COVID-19 and long COVID is the UK Office for National Statistics COVID Infection Survey^26^. This survey estimated that as of 7 April 2022, 1.7 million people living in private households in the UK (2.7% of the population) were experiencing symptoms persisting beyond 4 weeks from SARS-CoV-2 infection and with 70% experiencing symptoms beyond 12 weeks. Fatigue, shortness of breath, anosmia and difficulty concentrating were the main symptoms reported. The prevalence was greatest in females, those from more socioeconomically deprived areas, people working in health and social care and individuals living with health conditions and disabilities. Our analysis showed similar symptoms, including cognitive effects, as well as similar risk factors; however, we were unable to assess the association between occupational status and reporting of symptoms due to a lack of occupational data in UK primary care records.

Whittaker and colleagues undertook an analysis of 456,002 patients with COVID-19 in England using the Clinical Practice Research Datalink (CPRD) Aurum database to determine the rates of GP consultations for post-COVID-19 sequelae^27^. This analysis included both hospitalized and non-hospitalized patients and two control groups consisting of patients without COVID-19 and those with influenza before the pandemic. Patients with COVID-19 managed in the community were significantly more likely to consult for loss of taste or smell and other symptoms such as joint pain, anxiety, depression, abdominal pain and diarrhea at ≥ 4 weeks after infection compared to 12 months before infection. They also found that GP consultation rates for symptoms, prescriptions and healthcare use were mostly reduced in those who were managed in the community after the first COVID-19 vaccination dose; however, this study investigated only 23 symptoms based on the NICE 2020 guidelines^4^ on managing the long-term effects of COVID-19, whereas in our study, we investigated 115 symptoms derived from a systematic assessment of previous studies and discussions with patients with lived experience of long COVID and clinicians^11^.

We were unable to estimate the effect of vaccination and infection year on long COVID symptoms in our study due to the very short follow-up period among those vaccinated and infected in the year 2022 (median 8 (IQR 4–14) and 12 (7–16) days, respectively) compared to those unvaccinated and infected in the year 2021 (33 (16–77) and 64 (31–90) days, respectively). Furthermore, the majority (81%) of patients vaccinated before infection in our cohort were infected with SARS-CoV-2 within 2 weeks of vaccination, which would be before acquiring immunity from vaccination, thus restricting the validity of our data to assess the effects of vaccination on long COVID.

Further research is needed to estimate the prevalence of persistent symptoms associated with SARS-CoV-2 infection among patients presenting to primary care. Much of the symptom data in primary care records is held in free-text entries rather than as clinically coded data. Natural language processing could be used to leverage these textual data to gain more accurate estimates of the prevalence of these symptoms.

The 50 consolidated symptoms that were found to be associated with SARS-CoV-2, 12 weeks after infection in our study, were clustered into three phenotypes with varying risk factors. Further research is needed to confirm the identified clusters using prospective and routinely recorded patient-reported symptom data. This analysis would allow for assessment of whether clinical outcomes and the underlying pathophysiology differ between these subgroups and potentially develop targeted therapies for the different phenotypic subgroups. There is also a need to obtain patient-reported data on symptoms and assess the association between symptom burden, quality of life and work capability to ascertain which symptoms have the greatest impact on individuals. Finally, there is a need to understand the natural history of long COVID by assessing symptom burden serially over time in a population-representative cohort with a history of COVID-19 alongside a matched control population.

Infection with SARS-CoV-2 is independently associated with the reporting of 62 symptoms spanning multiple organ systems 12 weeks or longer after infection. A wide range of both sociodemographic and clinical factors are independently associated with the development of persistent symptoms. Additional research is needed to describe the natural history of long COVID and characterize symptom clusters, their pathophysiology and clinical outcomes. Further research is also needed to understand the health and social impacts of these persistent symptoms, to support patients living with long-term sequelae and to develop targeted treatments.

## Methods

### Study design and setting

This analysis was undertaken as part of the National Institute for Health and Care Research (NIHR) and UK Research and Innovation (UKRI)-funded Therapies for Long COVID in non-hospitalized individuals (TLC) study^28^. We conducted a population-based retrospective matched-cohort study between 31 January 2020 and 15 April 2021 using data from the Medicines and Healthcare products Regulatory Agency (MHRA) CPRD Aurum. CPRD Aurum is an anonymized database of primary care medical records of over 7 million actively registered patients in general practices that use the EMIS clinical information system^29^. It captures data on patient demographics, diagnoses, symptoms, prescriptions, referrals and tests. Structured data on diagnoses, symptoms and referrals are recorded using SNOMED CT coding terminology. Selection of SNOMED CT codes for data extraction was conducted by a team of clinical researchers using an inhouse developed software platform called Code Builder, with systematic searching of existing code lists, reference to the SNOMED CT terminology browser and through clinical knowledge and discussion. Data extraction was performed using the data extraction for epidemiological research (DExTER) tool for automated clinical epidemiological studies^30^.

### Participants

Patients aged 18 years and older with a minimum registration period of 12 months were included in the study. Practices were considered eligible 12 months after they were deemed to be providing research quality data. The cohort of patients with SARS-CoV-2 infection was defined as patients with a coded record of a positive RT–PCR test or antigen test result for SARS-CoV-2 and without a record of hospitalization 14 d before or 42 d after infection (within 28 d of infection with a ± 14-d grace period for clinical coding delays) in the primary care record. Their index date was assigned as the date of confirmation of SARS-CoV-2 infection. SNOMED CT codes for defining COVID-19 are listed in Supplementary Table 12a,b. For each patient infected with SARS-CoV-2, a pool of patients without a record of suspected or confirmed COVID-19 were selected from the database. These patients were assigned the same index date as the index date of the corresponding patient infected with SARS-CoV-2 to mitigate immortal time bias^31^.

### Propensity score matching

To control for confounding, each patient infected with SARS-CoV-2 was propensity score-matched with up to four patients with no recorded evidence of SARS-CoV-2 infection using a logistic regression model including the covariates listed in the covariates section below and a caliper width of 0.2. The SMD between patients infected with SARS-CoV-2 and patients with no recorded evidence of infection was reported for each variable before and after matching, and a variable with SMD > 0.1 after matching was considered to indicate imbalance in baseline characteristics. Kernel density plots were drawn for the two groups before and after matching to check the distribution of propensity scores.

### Outcomes and follow-up

We identified 115 relevant symptoms coded within primary care records (Supplementary Table 13) through a systematic review and meta-analysis of long COVID symptoms^11^, a scoping search of long COVID clinical assessment questionnaires, qualitative interviews with patients, a clinician survey and refinement of the symptom list using psychometric methods^32^. These were grouped into 15 domains: (1) breathing, (2) pain, (3) circulation, (4) fatigue, (5) cognitive health, (6) movement, (7) sleep, (8) ear, nose and throat, (9) stomach and digestion, (10) muscles and joints, (11) mental health, (12) hair, skin and nails, (13) eyes, (14) reproductive health and (15) other symptoms. SNOMED CT code lists for the symptoms are published on GitHub (https://github.com/AnuSub/LongCOVID_Symptoms_CodeList).

Our primary outcome definition of long COVID was pre-defined as the presence of at least one symptom included in the WHO case definition at ≥12 weeks after infection (Supplementary Table 13)^6^. Our secondary outcome definition of long COVID was derived post hoc as the presence of at least one symptom that was statistically associated with SARS-CoV-2 infection at ≥12 weeks after infection within this study (Supplementary Table 13).

The 115 symptoms were consolidated into 50 distinct symptoms to be included as categorical indicator variables for latent class analysis. This was carried out to avoid producing clusters of (1) commonly occurring symptoms that are not associated with COVID-19, (2) symptoms with mutually inclusive SNOMED CT codes (such as pain and chest pain) and (3) symptoms that commonly co-appear (such as nausea and vomiting).

Patients were followed up from the index date until the earliest of the following end points (patient exit date): (1) recording of symptoms of interest within the time interval studied, (2) death, (3) transfer out of practice, (4) end of general practice data and (5) study end date (15 April 2021). The follow-up period was split into three time periods from the index date: (1) the first 4 weeks (‘acute COVID-19’ among the cases), (2) 4–12 weeks (‘ongoing symptomatic COVID-19’) and (3) after 12 weeks (period of ‘post-COVID-19 condition’ or ‘long COVID’), in accordance with the current NICE guidelines on managing the long-term effects of COVID-19^4^.

### Covariates

We extracted data on demographic characteristics (age, sex, ethnic group, socioeconomic status and IMD), index week, BMI, smoking status and 87 chronic health conditions (Supplementary Table 1). We extracted data on 115 symptoms recorded in the period between 12 months and 3 months before the index date (at baseline). These variables were used to generate propensity scores for symptom burden to ensure that pre-existing health conditions and symptoms did not differ between the cohort of patients with and without recorded evidence of SARS-CoV-2 infection.

BMI was categorized as underweight (<18.5 kg m^−2^), healthy weight (18.5–24 kg m^−2^), overweight (25–29 kg m^−2^) and obese (≥30 kg m^−2^). Smoking status was categorized as never smoked, ex-smoker and current smoker. Ethnic group was categorized as either white, Asian (origin from India, Pakistan, China, Cambodia, Thailand, Vietnam, Malaysia, Sri Lanka, Nepal, Bangladesh, Japan or Taiwan), Black Afro-Caribbean, mixed or other ethnic group (native American, Middle Eastern and Polynesian origin). Missing data on ethnic group, socioeconomic status, BMI and smoking status were denoted by a ‘missing’ category within the corresponding variable.

### Statistical analysis

Continuous variables were summarized as mean and s.d. and categorical variables as frequencies and percentages. A series of Cox proportional hazards regression models were used to provide aHRs for each of the individual symptoms among patients with SARS-CoV-2 infection compared to patients with no recorded evidence of SARS-CoV-2 infection separately during the first 4, 4–12 and 12 weeks after the index date, with follow-up initiating from the index date, 4 weeks after the index date and 12 weeks after the index date, respectively. Patients with a minimum follow-up period of 4 and 12 weeks were included in the symptom outcome analyses at 4–12 and 12 weeks, respectively. Adjustments were made for age, sex, ethnic group, socioeconomic status, BMI, smoking status and the specified symptom recorded at baseline between 3 and 12 months before the index date. Multiple testing was accounted for by incorporating a Bonferroni correction to adjust the *P* value thresholds for statistical significance. Symptoms with statistically significant aHRs after Bonferroni correction in the period 12 weeks after the index date were presented in a forest plot. A post hoc-subgroup analysis was performed in a cohort of patients who were infected before and after 31 August 2020 (first and second surge of the pandemic) and propensity score-matched patients within the same sub-study period.

In a cohort restricted to patients with a positive RT–PCR or antigen test result for SARS-CoV-2 and a minimum of 12 weeks follow-up, unadjusted and adjusted Cox proportional hazards models were used to assess the association between the risk factors described in the covariates section and the primary (at least one of the symptoms in the WHO case definition for long COVID) and secondary (at least one of the symptoms statistically associated with SARS-CoV-2 infection) outcome definitions of long COVID. The median follow-up period and IQR were reported for patients within each risk factor strata. Hazard ratios were obtained by taking exponentiated coefficients from the Cox proportional hazards models, and we considered covariates with a *P* value <0.05 to be statistically significant.

A post hoc latent class analysis was performed on the 50 consolidated symptoms, and the model with the elbow point of fit for the Bayesian Information Criteria was considered optimal^33,34^. A multinomial logistic regression model was performed to identify the demographic features associated with each of the latent long COVID classes compared to patients without long COVID. All analyses were performed in Stata IC v.16 or R v.4.0.4.

### Ethical approval

CPRD obtains annual research ethics approval from the UK’s Health Research Authority Research Ethics Committee (East Midlands, Derby; reference no. 05/MRE04/87) to receive and supply patient data for public health research. Therefore, no additional ethics approval is required for observational studies using CPRD Aurum data for public health research, subject to individual research protocols meeting CPRD data governance requirements. The use of CPRD Aurum data for the study was approved by the CPRD Independent Scientific Advisory Committee (reference no. 21_000423).

### Reporting summary

Further information on research design is available in the Nature Research Reporting Summary linked to this article.

## Online content

Any methods, additional references, Nature Research reporting summaries, source data, extended data, supplementary information, acknowledgements, peer review information; details of author contributions and competing interests; and statements of data and code availability are available at 10.1038/s41591-022-01909-w.

## Supplementary information

Supplementary Information Supplementary Tables 1–13

Reporting Summary

## Data Availability

Access to anonymized patient data from CPRD is subject to a data sharing agreement containing detailed terms and conditions of use following protocol approval from the MHRA Independent Scientific Advisory Committee. This study-specific analyzable dataset is therefore not publicly available but can be requested from the corresponding author at K.Nirantharan@bham.ac.uk subject to research data governance approvals. Details about Independent Scientific Advisory Committee applications and data costs are available on the CPRD website (cprd.com).

## Code availability

Stata and R codes are available at https://github.com/AnuSub/Stata-and-R-codes
