# Supplementary material for: Symptoms and risk factors for long COVID in non-hospitalized adults
Source: Nat Med. 2022 Jul 25;28(8):1706–14. doi: 10.1038/s41591-022-01909-w (PMC9388369; doi:10.1038/s41591-022-01909-w)
Supplement: Supplementary file 1 — Supplementary Tables 1–13 [file 41591_2022_1909_MOESM1_ESM.pdf]

nature portfolio

https://doi.org/10.1038/s41591-022-01909-w

Supplementary information

Symptoms and risk factors for long COVID in non-hospitalized adults

In the format provided by the authors and unedited

Supplementary Table 1: Baseline comorbidities of patients infected with SARS CoV-2 and propensity matched comparator cohort of patients with no recorded evidence of SARS CoV-2 infection

Comorbidities	Cohort of patients infected with SARS CoV-2 (n=486,149) N (%)	Comparator cohort (n=1,944,580) N (%)	Standardised differences
**Cancer**	21249 (4.37)	83079 (4.27)	0.005
**Arrhythmia**	34811 (7.16)	136280 (7.01)	0.006
**AF**	9609 (1.98)	37397 (1.92)	0.004
**Hypertension**	73901 (15.20)	291389 (14.98)	0.006
**Heart Failure**	4342 (0.89)	16699 (0.86)	0.004
**IHD**	13654 (2.81)	53003 (2.73)	0.005
**Myocardial Infarction**	6761 (1.39)	26290 (1.35)	0.003
**Valvular Heart Disease**	6168 (1.27)	24019 (1.24)	0.003
**Cardiomyopathy**	1241 (0.26)	4854 (0.25)	0.001
**Congenital Heart Disease**	2968 (0.61)	11898 (0.61)	0.000
**PVD**	4622 (0.95)	18188 (0.94)	0.002
**Aortic Aneurysm**	966 (0.20)	3756 (0.19)	0.001
**TIA**	4383 (0.90)	16652 (0.86)	0.005
**Ischaemic Stroke**	2426 (0.50)	9353 (0.48)	0.003
**Haemorrhagic Stroke**	1404 (0.29)	5559 (0.29)	0.001
**Stroke Unspecified**	3865 (0.80)	14847 (0.76)	0.004
**Eczema**	94313 (19.40)	378604 (19.47)	0.002
**Psoriasis**	19881 (4.09)	79206 (4.07)	0.001
**Autoimmune Skin Conditions**	6561 (1.35)	26426 (1.36)	0.001
**Acne**	66474 (13.67)	267760 (13.77)	0.003
**Hay Fever**	87691 (18.04)	352090 (18.11)	0.002
**Chronic Sinusitis**	8403 (1.73)	33231 (1.71)	0.002
**Deafness**	5405 (1.11)	21363 (1.10)	0.001
**Blindness**	2263 (0.47)	8825 (0.45)	0.002
**Cataract**	16844 (3.46)	64397 (3.31)	0.008
**Glaucoma**	5132 (1.06)	19804 (1.02)	0.004
**AMD**	3518 (0.72)	13548 (0.70)	0.003
**Diabetic Retinopathy**	13029 (2.68)	51141 (2.63)	0.003
**Inflammatory Eye Disease**	8312 (1.71)	33090 (1.70)	0.001
**Peptic Ulcer**	6599 (1.36)	25960 (1.33)	0.002
**Inflammatory Bowel Disease**	4736 (0.97)	18880 (0.97)	0.000
**Irritable Bowel Syndrome**	34226 (7.04)	136963 (7.04)	0.000
**Hepatitis B**	1394 (0.29)	5535 (0.28)	0.000
**Hepatitis C**	840 (0.17)	3147 (0.16)	0.003
**Alcohol related chronic liver disease**	884 (0.18)	3421 (0.18)	0.001
**NAFLD**	6155 (1.27)	24477 (1.26)	0.001
**Chronic Liver Disease**	13573 (2.79)	53899 (2.77)	0.001
**Diverticular Disease**	14235 (2.93)	55938 (2.88)	0.003

**Coeliac Disease**	2125 (0.44)	8553 (0.44)	0.000
**Chronic Pancreatitis**	375 (0.08)	1450 (0.07)	0.001
**Endometriosis**	7050 (1.45)	28035 (1.44)	0.001
**PCOS**	11963 (2.46)	47699 (2.45)	0.001
**Low Haemoglobin**	26040 (5.36)	102708 (5.28)	0.003
**VTE**	9467 (1.95)	37297 (1.92)	0.002
**Coagulopathy**	5490 (1.13)	21880 (1.13)	0.000
**Pernicious Anaemia**	1263 (0.26)	4964 (0.26)	0.001
**Depression**	107392 (22.09)	428797 (22.05)	0.001
**Anxiety**	98849 (20.33)	395365 (20.33)	0.000
**Serious Mental Illness**	5420 (1.11)	21044 (1.08)	0.003
**Substance Misuse**	8523 (1.75)	32482 (1.67)	0.006
**Alcohol Misuse**	24940 (5.13)	99002 (5.09)	0.002
**ADHD**	2521 (0.52)	10089 (0.52)	0.000
**Eating Disorder**	4353 (0.90)	17365 (0.89)	0.000
**Learning Disability**	4449 (0.92)	17907 (0.92)	0.001
**Alzheimer's**	3978 (0.82)	13604 (0.70)	0.014
**Parkinson's disease**	1036 (0.21)	3982 (0.20)	0.002
**Vascular dementia**	2092 (0.43)	6617 (0.34)	0.015
**Migraine**	53881 (11.08)	215733 (11.09)	0.000
**Multiple Sclerosis**	1033 (0.21)	4203 (0.22)	0.001
**Epilepsy**	7718 (1.59)	30524 (1.57)	0.001
**Hemiplegia**	726 (0.15)	2882 (0.15)	0.000
**Chronic Fatigue Syndrome**	1711 (0.35)	6716 (0.35)	0.001
**Fibromyalgia**	5109 (1.05)	20364 (1.05)	0.000
**Cluster Headache**	1717 (0.35)	6847 (0.35)	0.000
**Osteoarthritis**	53694 (11.04)	211062 (10.85)	0.006
**Back pain**	6956 (1.43)	27386 (1.41)	0.002
**Fragility Fracture**	46608 (9.59)	186194 (9.58)	0.000
**Falls**	39765 (8.18)	155875 (8.02)	0.006
**Polymyalgia Rheumatica**	2187 (0.45)	8319 (0.43)	0.003
**Rheumatoid Arthritis**	4144 (0.85)	16252 (0.84)	0.002
**Raynaud's Disease**	5361 (1.10)	21244 (1.09)	0.001
**Sjogren's Syndrome**	541 (0.11)	2184 (0.11)	0.000
**SLE**	601 (0.12)	2386 (0.12)	0.000
**Systemic Sclerosis**	158 (0.03)	620 (0.03)	0.000
**Ankylosing Spondylitis**	780 (0.16)	3067 (0.16)	0.001
**Gout**	14039 (2.89)	55919 (2.88)	0.001
**CKD**	14526 (2.99)	56107 (2.89)	0.006
**Asthma**	97509 (20.06)	390401 (20.08)	0.000
**COPD**	10938 (2.25)	42308 (2.18)	0.005
**OSA**	6988 (1.44)	28024 (1.44)	0.000
**Other Pulmonary Disease**	2631 (0.54)	10144 (0.52)	0.003
**Hypothyroidism**	21065 (4.33)	83404 (4.29)	0.002
**Type 1 diabetes**	2831 (0.58)	11150 (0.57)	0.001
**Type 2 diabetes**	32457 (6.68)	128050 (6.58)	0.004

**AIDS**	932 (0.19)	3731 (0.19)	0.000
**BPH**	6765 (1.39)	26293 (1.35)	0.003
**Erectile Dysfunction**	21336 (4.39)	84805 (4.36)	0.001

AF= Atrial Fibrillation, IHD=Ischaemic Heart Disease, PVD=Peripheral Vascular Disease, TIA=Transient Ischaemic Attack, AMD=Age-related Macular Degeneration, NAFLD=Non-Alcoholic Fatty Liver Disease, PCOS=Poly Cystic Ovarian Syndrome, VTE=Venous Thromboembolism, ADHD=Attention Deficit Hyperactivity Disorder, SLE=Systemic Lupus Erythematosus, CKD=Chronic Kidney Disease, COPD=Chronic Obstructive Pulmonary Disease, OSA=Obstructive Sleep Apnoea, AIDS=Acquired Immune Deficiency Syndrome, BPH=Benign Prostatic Hyperplasia; standardised difference of less than 0.1 indicates a relatively small imbalance

Supplementary Table 2: Recording of symptoms between 3 and 12 months prior to index date comparing between patients infected with SARS CoV-2 and propensity matched comparator cohort of patients with no recorded evidence of SARS CoV-2 infection

Domain	Symptom	Cohort of patients infected with SARS CoV-2 (n=486,149) N (%)	Comparator cohort (n=1,944,580) N (%)	Adjusted OR (95% CI)*
Breathing	Orthopnoea	38 (0.01)	124 (0.01)	1.21 (0.84-1.75)
	Paroxysmal nocturnal dyspnoea	26 (0.01)	91 (0.00)	1.11 (0.72-1.72)
	Shortness of breath	8132 (1.67)	31420 (1.62)	1.01 (0.99-1.04)
	Shortness of breath at rest	257 (0.05)	945 (0.05)	1.05 (0.92-1.21)
	Shortness of breath on exertion	7096 (1.46)	27472 (1.41)	1.01 (0.98-1.04)
	Tachypnoea	39 (0.01)	157 (0.01)	0.98 (0.69-1.39)
	Wheezing	2122 (0.44)	8224 (0.42)	1.02 (0.97-1.07)
Pain	Chest pain	8698 (1.79)	34551 (1.78)	0.99 (0.97-1.01)
	Neuropathic pain	977 (0.20)	3943 (0.20)	0.97 (0.91-1.04)
	Pain	73174 (15.05)	290921 (14.96)	0.99 (0.98-1.00)
	Pleuritic chest pain	323 (0.07)	1245 (0.06)	1.03 (0.91-1.16)
Circulation	Cold extremities	70 (0.01)	298 (0.02)	0.92 (0.71-1.20)
	Limb swelling	3308 (0.68)	12855 (0.66)	0.99 (0.96-1.03)
	Orthostatic hypotension	445 (0.09)	1472 (0.08)	1.16 (1.04-1.29)
	Palpitations	2577 (0.53)	10195 (0.52)	1.00 (0.96-1.05)
	Presyncope	181 (0.04)	738 (0.04)	0.97 (0.83-1.15)
	Tachycardia	646 (0.13)	2576 (0.13)	0.99 (0.91-1.08)
Fatigue	Fatigue	6327 (1.30)	24977 (1.28)	1.00 (0.98-1.03)
	Post exertional fatigue	0 (0.00)	0 (0.00)	
Cognitive health	Amnesia	1087 (0.22)	4132 (0.21)	1.00 (0.93-1.07)
	Brain fog	1494 (0.31)	5873 (0.30)	1.00 (0.94-1.06)
	Difficulty understanding	7 (0.00)	22 (0.00)	1.21 (0.52-2.84)
	Dysarthria	68 (0.01)	266 (0.01)	0.98 (0.75-1.28)
	Dysphasia	34 (0.01)	142 (0.01)	0.92 (0.63-1.34)
	Reading difficulty	1 (0.00)	2 (0.00)	1.89 (0.17-20.87)
Movement	Apraxia	61 (0.01)	242 (0.01)	0.98 (0.74-1.30)
	Balance difficulty	355 (0.07)	1325 (0.07)	1.02 (0.91-1.15)
	Tremors	434 (0.09)	1669 (0.09)	1.02 (0.92-1.14)
Sleep	Excessive sleep	41 (0.01)	145 (0.01)	1.12 (0.79-1.59)
	Insomnia	3650 (0.75)	14352 (0.74)	1.00 (0.97-1.04)
Ear, nose and throat	Anosmia	609 (0.13)	2307 (0.12)	1.05 (0.96-1.14)
	Cough	18224 (3.75)	71056 (3.65)	1.00 (0.99-1.02)
	Dysguesia	480 (0.10)	1827 (0.09)	1.04 (0.94-1.15)

	**Dysphagia**	1296 (0.27)	4893 (0.25)	1.03 (0.96-1.09)
	**Ear pain**	3895 (0.80)	15527 (0.80)	1.00 (0.96-1.03)
	**Hearing loss**	28 (0.01)	105 (0.01)	1.04 (0.68-1.58)
	**Hoarse voice**	616 (0.13)	2368 (0.12)	1.02 (0.94-1.12)
	**Hyperacusis**	15 (0.00)	62 (0.00)	0.95 (0.54-1.67)
	**Nasal congestion**	2352 (0.48)	9249 (0.48)	1.01 (0.96-1.05)
	**Phlegm**	3535 (0.73)	13744 (0.71)	1.00 (0.97-1.04)
	**Sneezing**	79 (0.02)	292 (0.02)	1.07 (0.83-1.37)
	**Sore throat**	11196 (2.30)	42052 (2.16)	1.06 (1.04-1.09)
	**Tinnitus**	1869 (0.38)	7463 (0.38)	0.99 (0.94-1.04)
**Stomach and digestion**	**Abdominal pain**	12486 (2.57)	49419 (2.54)	1.00 (0.98-1.02)
	**Bloating**	1210 (0.25)	4637 (0.24)	1.03 (0.97-1.10)
	**Bowel incontinence**	765 (0.16)	2424 (0.12)	1.20 (1.10-1.30)
	**Constipation**	3892 (0.80)	15154 (0.78)	1.00 (0.96-1.04)
	**Diarrhoea**	4205 (0.86)	16273 (0.84)	1.01 (0.98-1.05)
	**Gastric reflux**	3961 (0.81)	15656 (0.81)	1.00 (0.97-1.04)
	**gastritis**	1804 (0.37)	7161 (0.37)	0.99 (0.94-1.04)
	**Nausea**	1389 (0.29)	5374 (0.28)	1.02 (0.97-1.09)
	**Vomiting**	1678 (0.35)	6552 (0.34)	1.01 (0.95-1.06)
	**Weight gain**	384 (0.08)	1525 (0.08)	1.00 (0.90-1.12)
	**Weight loss**	1071 (0.22)	4057 (0.21)	1.01 (0.94-1.08)
**Muscles and joints**	**Asthenia**	239 (0.05)	910 (0.05)	1.01 (0.88-1.17)
	**Joint pain**	3265 (0.67)	12820 (0.66)	1.00 (0.97-1.04)
	**Joint stiffness**	36 (0.01)	152 (0.01)	0.94 (0.65-1.35)
	**Muscle cramping**	905 (0.19)	3498 (0.18)	1.02 (0.95-1.10)
	**Muscle pain**	1499 (0.31)	5854 (0.30)	1.02 (0.96-1.08)
	**Muscle twitch**	5 (0.00)	19 (0.00)	1.02 (0.38-2.72)
	**Paraesthesia**	1782 (0.37)	6942 (0.36)	1.02 (0.96-1.07)
**Mental health**	**Anhedonia**	1555 (0.32)	6204 (0.32)	0.98 (0.93-1.04)
	**Anorexia**	891 (0.18)	3366 (0.17)	1.02 (0.95-1.10)
	**Anxiety**	18670 (3.84)	74351 (3.82)	1.00 (0.99-1.02)
	**Anxiety and depression**	8190 (1.68)	32806 (1.69)	1.00 (0.98-1.03)
	**Depression**	18828 (3.87)	75291 (3.87)	1.00 (0.98-1.02)
	**Increased appetite**	39 (0.01)	158 (0.01)	0.98 (0.69-1.39)
	**loneliness**	114 (0.02)	443 (0.02)	1.00 (0.81-1.23)
	**Mood swings**	281 (0.06)	1093 (0.06)	1.01 (0.89-1.16)
	**Post-traumatic stress disorder**	116 (0.02)	455 (0.02)	1.04 (0.85-1.28)
**Hair, skin and nails**	**Dry and scaly skin**	684 (0.14)	2660 (0.14)	1.00 (0.92-1.09)
	**Hair loss**	1254 (0.26)	4892 (0.25)	1.03 (0.96-1.09)
	**Hives**	1075 (0.22)	4167 (0.21)	1.02 (0.96-1.09)
	**Itchy skin**	1815 (0.37)	7108 (0.37)	1.00 (0.95-1.05)
	**Nail changes**	1636 (0.34)	6593 (0.34)	0.98 (0.93-1.04)
	**Purpura**	82 (0.02)	329 (0.02)	0.97 (0.76-1.24)
	**Rash**	7979 (1.64)	31669 (1.63)	0.99 (0.97-1.02)
**Eyes**	**Diplopia**	325 (0.07)	1225 (0.06)	1.05 (0.93-1.18)

	**Dry eye**	906 (0.19)	3641 (0.19)	0.97 (0.90-1.05)
	**Eye pain**	240 (0.05)	969 (0.05)	0.98 (0.85-1.13)
	**Flashing lights and floaters**	140 (0.03)	542 (0.03)	1.03 (0.86-1.24)
	**Itchy eyes**	128 (0.03)	444 (0.02)	1.13 (0.93-1.38)
	**Photophobia**	53 (0.01)	197 (0.01)	1.08 (0.80-1.47)
	**Red eye**	2520 (0.52)	9882 (0.51)	0.99 (0.95-1.04)
	**Watery eyes**	134 (0.03)	523 (0.03)	1.00 (0.83-1.21)
**Reproductive health**	**Ejaculation difficulty**	89 (0.02)	329 (0.02)	1.07 (0.84-1.35)
	**Erectile dysfunction**	1543 (0.32)	6152 (0.32)	0.97 (0.92-1.03)
	**Menorrhagia**	2623 (0.54)	10374 (0.53)	1.01 (0.97-1.06)
	**Menstrual changes**	2532 (0.52)	9902 (0.51)	1.03 (0.99-1.08)
	**Premenstrual syndrome**	255 (0.05)	1044 (0.05)	0.98 (0.86-1.13)
	**Vaginal discharge**	4134 (0.85)	16480 (0.85)	1.01 (0.97-1.04)
	**Vaginal dryness**	191 (0.04)	797 (0.04)	0.94 (0.80-1.10)
	**Anorgasm**	3 (0.00)	8 (0.00)	1.62 (0.43-6.10)
	**Reduced libido**	136 (0.03)	520 (0.03)	1.04 (0.86-1.26)
**Other symptoms**	**Allergies**	4908 (1.01)	19148 (0.98)	1.02 (0.99-1.05)
	**Angioedema**	517 (0.11)	1953 (0.10)	1.05 (0.95-1.15)
	**Body ache**	755 (0.16)	2883 (0.15)	1.03 (0.95-1.12)
	**Chills and shivering**	121 (0.02)	411 (0.02)	
	**Dizziness**	3480 (0.72)	13754 (0.71)	0.99 (0.96-1.03)
	**Dry mouth**	246 (0.05)	950 (0.05)	1.01 (0.87-1.16)
	**Fever**	3315 (0.68)	12481 (0.64)	1.04 (1.00-1.09)
	**Haemoptysis**	322 (0.07)	1260 (0.06)	1.00 (0.89-1.14)
	**Hallucinations**	243 (0.05)	928 (0.05)	1.01 (0.88-1.16)
	**Headache**	12361 (2.54)	48501 (2.49)	1.01 (0.99-1.03)
	**Hot flushes**	789 (0.16)	3182 (0.16)	0.97 (0.90-1.05)
	**Mouth ulcer**	628 (0.13)	2483 (0.13)	1.00 (0.91-1.09)
	**Neurasthenia**	19 (0.00)	66 (0.00)	1.18 (0.71-1.97)
	**Polydipsia**	104 (0.02)	418 (0.02)	0.98 (0.79-1.22)
	**Polyuria**	2783 (0.57)	10927 (0.56)	1.00 (0.96-1.04)
	**Seizures**	695 (0.14)	2659 (0.14)	1.03 (0.94-1.12)
	**Sweating**	846 (0.17)	3386 (0.17)	0.99 (0.92-1.07)
	**Swelling of lymph nodes**	1406 (0.29)	5489 (0.28)	1.02 (0.97-1.09)
	**Urinary incontinence**	2190 (0.45)	8004 (0.41)	1.05 (1.00-1.11)
	**Urinary retention**	442 (0.09)	1719 (0.09)	0.98 (0.89-1.09)
	**Vertigo**	2220 (0.46)	8715 (0.45)	1.00 (0.96-1.05)

*Adjusted for age, sex, body mass index, smoking status, ethnicity and socioeconomic deprivation

Supplementary Table 3A: Recording of symptoms after twelve weeks from index date comparing between patients infected with SARS CoV-2 and propensity matched comparator cohort of patients with no recorded evidence of SARS CoV-2 infection

Domain	Symptom	Cohort of patients infected with SARS CoV-2 (n=384,137) N (%)	Comparator cohort (n=1,501,689) N (%)	Adjusted HR (95% CI)*
**Composite Symptom outcome**	**Composite of symptoms in the definition for Long COVID from WHO report**	20864 (5.43)	65293 (4.35)	1.26 (1.25-1.28)
	**Composite of symptoms that were significantly associated with COVID-19 exposure after twelve weeks from index date**	35705 (9.29)	110737 (7.37)	1.29 (1.28-1.31)
**Breathing**	**Orthopnoea**	10 (0.00)	41 (0.00)	1.07 (0.54-2.15)
	**Paroxysmal nocturnal dyspnoea**	3 (0.00)	23 (0.00)	0.60 (0.18-1.99)
	**Shortness of breath**	1615 (0.42)	5398 (0.36)	1.31 (1.24-1.38)
	**Shortness of breath at rest**	51 (0.01)	105 (0.01)	2.20 (1.57-3.08)
	**Shortness of breath on exertion**	1406 (0.37)	4905 (0.33)	1.26 (1.18-1.33)
	**Tachypnoea**	4 (0.00)	16 (0.00)	1.00 (0.33-2.99)
	**Wheezing**	410 (0.11)	1164 (0.08)	1.42 (1.27-1.59)
**Pain**	**Chest pain**	1821 (0.47)	5096 (0.34)	1.42 (1.35-1.50)
	**Neuropathic pain**	76 (0.02)	345 (0.02)	0.86 (0.67-1.11)
	**Pain**	14958 (3.89)	47694 (3.18)	1.25 (1.23-1.27)
	**Pleuritic chest pain**	73 (0.02)	157 (0.01)	1.86 (1.41-2.46)
**Circulation**	**Cold extremities**	30 (0.01)	87 (0.01)	1.40 (0.92-2.12)
	**Limb swelling**	674 (0.18)	2398 (0.16)	1.25 (1.15-1.36)
	**Orthostatic hypotension**	95 (0.02)	342 (0.02)	1.24 (0.99-1.56)
	**Palpitations**	682 (0.18)	1738 (0.12)	1.53 (1.40-1.67)
	**Presyncope**	27 (0.01)	169 (0.01)	0.70 (0.47-1.06)
	**Tachycardia**	172 (0.04)	481 (0.03)	1.44 (1.21-1.72)
**Fatigue**	**Fatigue**	1754 (0.46)	3559 (0.24)	1.92 (1.81-2.03)
	**Post exertional fatigue**	0 (0.00)	0 (0.00)	
**Cognitive health**	**Amnesia**	201 (0.05)	839 (0.06)	1.05 (0.90-1.22)
	**Brain fog**	221 (0.06)	627 (0.04)	1.37 (1.17-1.59)
	**Difficulty understanding**	4 (0.00)	12 (0.00)	1.27 (0.41-3.94)
	**Dysarthria**	19 (0.00)	67 (0.00)	1.28 (0.77-2.13)
	**Dysphasia**	11 (0.00)	33 (0.00)	1.43 (0.72-2.85)
	**Reading difficulty**	0 (0.00)	0 (0.00)	
**Movement**	**Apraxia**	15 (0.00)	55 (0.00)	1.12 (0.63-1.99)

	Balance difficulty	61 (0.02)	262 (0.02)	1.05 (0.80-1.39)
	Tremors	104 (0.03)	395 (0.03)	1.12 (0.90-1.39)
Sleep	Excessive sleep	6 (0.00)	37 (0.00)	0.65 (0.27-1.54)
	Insomnia	746 (0.19)	2190 (0.15)	1.34 (1.23-1.46)
Ear, nose and throat	Anosmia	153 (0.04)	95 (0.01)	6.49 (5.02-8.39)
	Cough	2127 (0.55)	6126 (0.41)	1.44 (1.37-1.51)
	Dysgeusia	42 (0.01)	180 (0.01)	0.97 (0.69-1.36)
	Dysphagia	334 (0.09)	864 (0.06)	1.60 (1.41-1.82)
	Ear pain	606 (0.16)	2044 (0.14)	1.16 (1.06-1.27)
	Hearing loss	11 (0.00)	33 (0.00)	1.52 (0.77-3.02)
	Hoarse voice	125 (0.03)	295 (0.02)	1.78 (1.44-2.20)
	Hyperacusis	2 (0.00)	11 (0.00)	0.67 (0.15-3.05)
	Nasal congestion	396 (0.10)	1165 (0.08)	1.34 (1.20-1.50)
	Phlegm	429 (0.11)	1390 (0.09)	1.33 (1.19-1.48)
	Sneezing	14 (0.00)	20 (0.00)	2.77 (1.40-5.50)
	Sore throat	359 (0.09)	1365 (0.09)	1.02 (0.91-1.15)
	Tinnitus	195 (0.05)	824 (0.05)	0.92 (0.78-1.07)
Stomach and digestion	Abdominal pain	2442 (0.64)	7818 (0.52)	1.21 (1.16-1.27)
	Bloating	270 (0.07)	814 (0.05)	1.31 (1.14-1.50)
	Bowel incontinence	185 (0.05)	432 (0.03)	1.58 (1.33-1.88)
	Constipation	829 (0.22)	2856 (0.19)	1.20 (1.11-1.30)
	Diarrhoea	693 (0.18)	2240 (0.15)	1.29 (1.19-1.41)
	Gastric reflux	736 (0.19)	2778 (0.18)	1.05 (0.96-1.13)
	gastritis	333 (0.09)	1112 (0.07)	1.20 (1.07-1.36)
	Nausea	309 (0.08)	891 (0.06)	1.37 (1.20-1.56)
	Vomiting	369 (0.10)	995 (0.07)	1.48 (1.31-1.67)
	Weight gain	96 (0.02)	292 (0.02)	1.27 (1.01-1.61)
	Weight loss	278 (0.07)	854 (0.06)	1.34 (1.17-1.53)
Muscles and joints	Asthenia	57 (0.01)	163 (0.01)	1.45 (1.07-1.96)
	Joint pain	659 (0.17)	2133 (0.14)	1.23 (1.13-1.35)
	Joint stiffness	10 (0.00)	28 (0.00)	1.51 (0.73-3.12)
	Muscle cramping	152 (0.04)	574 (0.04)	1.08 (0.90-1.29)
	Muscle pain	310 (0.08)	1082 (0.07)	1.13 (0.99-1.28)
	Muscle twitch	3 (0.00)	3 (0.00)	3.37 (0.68-16.82)
	Paraesthesia	418 (0.11)	1241 (0.08)	1.34 (1.20-1.50)
Mental health	Anhedonia	209 (0.05)	598 (0.04)	1.36 (1.16-1.59)
	Anorexia	174 (0.05)	566 (0.04)	1.28 (1.08-1.52)
	Anxiety	3732 (0.97)	12583 (0.84)	1.12 (1.08-1.16)
	Anxiety and depression	1705 (0.44)	6182 (0.41)	1.05 (1.00-1.11)
	Depression	3441 (0.90)	12117 (0.81)	1.09 (1.05-1.13)
	Increased appetite	5 (0.00)	30 (0.00)	0.68 (0.26-1.75)
	loneliness	7 (0.00)	59 (0.00)	0.49 (0.22-1.06)
	Mood swings	60 (0.02)	199 (0.01)	1.14 (0.86-1.53)
	Post-traumatic stress disorder	27 (0.01)	137 (0.01)	0.76 (0.50-1.15)
	Dry and scaly skin	186 (0.05)	577 (0.04)	1.30 (1.10-1.53)

**Hair, skin and nails**	**Hair loss**	869 (0.23)	825 (0.05)	3.99 (3.63-4.39)
	**Hives**	168 (0.04)	545 (0.04)	1.18 (0.99-1.40)
	**Itchy skin**	358 (0.09)	1098 (0.07)	1.34 (1.19-1.51)
	**Nail changes**	298 (0.08)	970 (0.06)	1.20 (1.05-1.37)
	**Purpura**	15 (0.00)	57 (0.00)	1.06 (0.60-1.87)
	**Rash**	1495 (0.39)	4790 (0.32)	1.24 (1.17-1.32)
**Eyes**	**Diplopia**	77 (0.02)	244 (0.02)	1.29 (1.00-1.67)
	**Dry eye**	217 (0.06)	700 (0.05)	1.28 (1.10-1.49)
	**Eye pain**	49 (0.01)	157 (0.01)	1.22 (0.88-1.68)
	**Flashing lights and floaters**	35 (0.01)	140 (0.01)	1.02 (0.70-1.48)
	**Itchy eyes**	21 (0.01)	50 (0.00)	1.68 (1.01-2.79)
	**Photophobia**	12 (0.00)	22 (0.00)	2.07 (1.02-4.19)
	**Red eye**	358 (0.09)	1089 (0.07)	1.34 (1.19-1.51)
	**Watery eyes**	16 (0.00)	53 (0.00)	1.25 (0.71-2.18)
**Reproductive health**	**Ejaculation difficulty**	28 (0.01)	39 (0.00)	2.63 (1.61-4.28)
	**Erectile dysfunction**	284 (0.07)	921 (0.06)	1.26 (1.10-1.44)
	**Menorrhagia**	490 (0.13)	1527 (0.10)	1.18 (1.07-1.31)
	**Menstrual changes**	525 (0.14)	1916 (0.13)	0.99 (0.90-1.09)
	**Premenstrual syndrome**	43 (0.01)	182 (0.01)	0.86 (0.61-1.19)
	**Vaginal discharge**	987 (0.26)	2894 (0.19)	1.26 (1.17-1.35)
	**Vaginal dryness**	34 (0.01)	133 (0.01)	1.03 (0.71-1.51)
	**Anorgasm**	0 (0.00)	2 (0.00)	
	**Reduced libido**	42 (0.01)	69 (0.00)	2.36 (1.61-3.47)
**Other symptoms**	**Allergies**	694 (0.18)	2042 (0.14)	1.30 (1.19-1.42)
	**Angioedema**	89 (0.02)	305 (0.02)	1.15 (0.91-1.46)
	**Body ache**	162 (0.04)	431 (0.03)	1.46 (1.22-1.75)
	**Chills and shivering**	15 (0.00)	46 (0.00)	1.36 (0.76-2.44)
	**Dizziness**	753 (0.20)	2527 (0.17)	1.23 (1.13-1.33)
	**Dry mouth**	67 (0.02)	179 (0.01)	1.56 (1.18-2.07)
	**Fever**	357 (0.09)	839 (0.06)	1.75 (1.54-1.98)
	**Haemoptysis**	64 (0.02)	192 (0.01)	1.39 (1.05-1.85)
	**Hallucinations**	44 (0.01)	181 (0.01)	1.01 (0.72-1.40)
	**Headache**	2654 (0.69)	7812 (0.52)	1.30 (1.24-1.35)
	**Hot flushes**	153 (0.04)	407 (0.03)	1.52 (1.27-1.84)
	**Mouth ulcer**	140 (0.04)	335 (0.02)	1.60 (1.32-1.95)
	**Neurasthenia**	2 (0.00)	25 (0.00)	0.30 (0.07-1.28)
	**Polydipsia**	27 (0.01)	77 (0.01)	1.36 (0.88-2.11)
	**Polyuria**	512 (0.13)	1664 (0.11)	1.27 (1.15-1.40)
	**Seizures**	132 (0.03)	484 (0.03)	1.05 (0.87-1.28)
	**Sweating**	158 (0.04)	562 (0.04)	1.09 (0.91-1.30)
	**Swelling of lymph nodes**	135 (0.04)	429 (0.03)	1.18 (0.97-1.44)
	**Urinary incontinence**	505 (0.13)	1520 (0.10)	1.37 (1.24-1.52)
	**Urinary retention**	113 (0.03)	312 (0.02)	1.59 (1.28-1.97)
	**Vertigo**	428 (0.11)	1536 (0.10)	1.15 (1.03-1.28)

*Adjusted for age, sex, body mass index, smoking status, ethnicity, socioeconomic deprivation and symptom recorded at baseline between 3 and 12 months prior to index date

Supplementary Table 3B: Number of recorded of symptoms after twelve weeks from the index date comparing patients infected with SARS CoV-2 and propensity matched comparator cohort of patients with no recorded evidence of SARS CoV-2 infection

Number of recorded symptoms after twelve weeks from the index date	Cohort of patients infected with SARS CoV-2 (n=384,137) N (%)	Comparator cohort (n=1,501,689) N (%)
**0**	330,044 (85.92)	1,328,051 (88.44)
**1**	21,473 (5.59)	70,562 (4.70)
**2**	13,808 (3.59)	43,334 (2.89)
**3**	10,367 (2.70)	32,652 (2.17)
**4**	5,378 (1.40)	17,693 (1.18)
**5**	1,752 (0.46)	5,541 (0.37)
**6 or more**	1,315 (0.34)	3,856 (0.26)

Supplementary Table 4: Recording of symptoms during the first four weeks after index date comparing between patients infected with SARS CoV-2 and propensity matched comparator cohort of patients no recorded evidence of SARS CoV-2 infection

Domain	Symptom	Cohort of patients infected with SARS CoV-2 (n=486,149) N (%)	Comparator cohort (n=1,944,580) N (%)	Adjusted HR (95% CI)*
Breathing	Orthopnoea	11 (0.00)	23 (0.00)	1.50 (0.69-3.28)
	Paroxysmal nocturnal dyspnoea	3 (0.00)	2 (0.00)	6.20 (1.03-37.18)
	Shortness of breath	5564 (1.14)	3245 (0.17)	6.03 (5.76-6.31)
	Shortness of breath at rest	440 (0.09)	78 (0.00)	19.75 (15.35-25.40)
	Shortness of breath on exertion	4507 (0.93)	2899 (0.15)	5.41 (5.15-5.68)
	Tachypnoea	23 (0.00)	11 (0.00)	6.91 (3.28-14.53)
	Wheezing	352 (0.07)	827 (0.04)	1.56 (1.37-1.78)
Pain	Chest pain	2657 (0.55)	3400 (0.17)	2.91 (2.76-3.07)
	Neuropathic pain	85 (0.02)	325 (0.02)	1.04 (0.81-1.33)
	Pain	16645 (3.42)	37941 (1.95)	1.63 (1.60-1.66)
	Pleuritic chest pain	224 (0.05)	113 (0.01)	7.36 (5.82-9.31)
Circulation	Cold extremities	14 (0.00)	53 (0.00)	1.05 (0.57-1.93)
	Limb swelling	341 (0.07)	1274 (0.07)	1.05 (0.92-1.18)
	Orthostatic hypotension	163 (0.03)	167 (0.01)	2.84 (2.24-3.60)
	Palpitations	523 (0.11)	1301 (0.07)	1.58 (1.42-1.76)
	Presyncope	33 (0.01)	103 (0.01)	1.20 (0.79-1.82)
	Tachycardia	262 (0.05)	260 (0.01)	3.86 (3.23-4.61)
Fatigue	Fatigue	3410 (0.70)	2274 (0.12)	5.65 (5.35-5.97)
	Post exertional fatigue	0 (0.00)	0 (0.00)	
Cognitive health	Amnesia	123 (0.03)	390 (0.02)	1.14 (0.92-1.41)
	Brain fog	134 (0.03)	503 (0.03)	1.05 (0.87-1.28)
	Difficulty understanding	3 (0.00)	4 (0.00)	2.95 (0.66-13.25)
	Dysarthria	18 (0.00)	28 (0.00)	1.27 (0.60-2.70)
	Dysphasia	5 (0.00)	18 (0.00)	0.42 (0.10-1.84)
	Reading difficulty	0 (0.00)	0 (0.00)	
Movement	Apraxia	10 (0.00)	41 (0.00)	0.96 (0.48-1.92)
	Balance difficulty	35 (0.01)	129 (0.01)	0.91 (0.61-1.35)
	Tremors	75 (0.02)	254 (0.01)	1.12 (0.86-1.47)
Sleep	Excessive sleep	2 (0.00)	15 (0.00)	0.55 (0.13-2.42)
	Insomnia	617 (0.13)	1775 (0.09)	1.38 (1.26-1.52)
Ear, nose and throat	Anosmia	3303 (0.68)	106 (0.01)	107.42 (88.16-130.89)
	Cough	11999 (2.47)	4179 (0.21)	9.96 (9.60-10.33)
	Dysgeusia	2726 (0.56)	90 (0.00)	111.13 (89.05-138.70)

	**Dysphagia**	243 (0.05)	532 (0.03)	1.64 (1.39-1.92)
	**Ear pain**	610 (0.13)	1617 (0.08)	1.46 (1.32-1.60)
	**Hearing loss**	0 (0.00)	14 (0.00)	
	**Hoarse voice**	63 (0.01)	158 (0.01)	1.41 (1.03-1.93)
	**Hyperacusis**	2 (0.00)	5 (0.00)	1.64 (0.32-8.45)
	**Nasal congestion**	333 (0.07)	772 (0.04)	1.65 (1.45-1.89)
	**Phlegm**	2260 (0.46)	891 (0.05)	8.64 (7.96-9.37)
	**Sneezing**	7 (0.00)	21 (0.00)	1.33 (0.56-3.12)
	**Sore throat**	2441 (0.50)	1852 (0.10)	4.66 (4.36-4.97)
	**Tinnitus**	138 (0.03)	636 (0.03)	0.84 (0.70-1.02)
**Stomach and digestion**	**Abdominal pain**	1602 (0.33)	6057 (0.31)	0.99 (0.94-1.05)
	**Bloating**	109 (0.02)	573 (0.03)	0.73 (0.59-0.90)
	**Bowel incontinence**	83 (0.02)	239 (0.01)	1.15 (0.88-1.49)
	**Constipation**	759 (0.16)	1808 (0.09)	1.55 (1.42-1.70)
	**Diarrhoea**	1245 (0.26)	1582 (0.08)	2.95 (2.73-3.19)
	**Gastric reflux**	564 (0.12)	2003 (0.10)	1.10 (1.00-1.21)
	**gastritis**	273 (0.06)	788 (0.04)	1.30 (1.12-1.50)
	**Nausea**	612 (0.13)	662 (0.03)	3.58 (3.19-4.01)
	**Vomiting**	573 (0.12)	682 (0.04)	2.99 (2.66-3.36)
	**Weight gain**	37 (0.01)	200 (0.01)	0.78 (0.55-1.11)
	**Weight loss**	229 (0.05)	520 (0.03)	1.66 (1.41-1.95)
**Muscles and joints**	**Asthenia**	123 (0.03)	79 (0.00)	5.47 (4.08-7.34)
	**Joint pain**	274 (0.06)	1381 (0.07)	0.79 (0.69-0.90)
	**Joint stiffness**	0 (0.00)	22 (0.00)	
	**Muscle cramping**	82 (0.02)	384 (0.02)	0.81 (0.64-1.04)
	**Muscle pain**	1430 (0.29)	859 (0.04)	6.14 (5.62-6.70)
	**Muscle twitch**	0 (0.00)	2 (0.00)	
	**Paraesthesia**	205 (0.04)	798 (0.04)	1.03 (0.88-1.21)
**Mental health**	**Anhedonia**	123 (0.03)	487 (0.03)	1.02 (0.83-1.24)
	**Anorexia**	461 (0.09)	358 (0.02)	4.48 (3.88-5.18)
	**Anxiety**	3264 (0.67)	13138 (0.68)	0.97 (0.93-1.00)
	**Anxiety and depression**	1464 (0.30)	6833 (0.35)	0.84 (0.79-0.89)
	**Depression**	2844 (0.59)	12740 (0.66)	0.87 (0.84-0.91)
	**Increased appetite**	3 (0.00)	18 (0.00)	0.67 (0.20-2.26)
	**loneliness**	13 (0.00)	49 (0.00)	1.18 (0.64-2.18)
	**Mood swings**	18 (0.00)	125 (0.01)	0.52 (0.31-0.87)
	**Post-traumatic stress disorder**	26 (0.01)	126 (0.01)	0.75 (0.48-1.18)
**Hair, skin and nails**	**Dry and scaly skin**	81 (0.02)	287 (0.01)	1.14 (0.88-1.46)
	**Hair loss**	136 (0.03)	579 (0.03)	0.91 (0.75-1.10)
	**Hives**	183 (0.04)	458 (0.02)	1.49 (1.25-1.78)
	**Itchy skin**	228 (0.05)	764 (0.04)	1.19 (1.02-1.39)
	**Nail changes**	133 (0.03)	623 (0.03)	0.84 (0.69-1.01)
	**Purpura**	10 (0.00)	34 (0.00)	1.38 (0.67-2.83)
	**Rash**	1121 (0.23)	3226 (0.17)	1.37 (1.27-1.47)
**Eyes**	**Diplopia**	51 (0.01)	127 (0.01)	1.56 (1.12-2.18)

	**Dry eye**	73 (0.02)	420 (0.02)	0.70 (0.54-0.89)
	**Eye pain**	34 (0.01)	97 (0.00)	1.30 (0.87-1.95)
	**Flashing lights and floaters**	17 (0.00)	66 (0.00)	0.99 (0.57-1.71)
	**Itchy eyes**	11 (0.00)	35 (0.00)	1.32 (0.67-2.62)
	**Photophobia**	15 (0.00)	22 (0.00)	2.61 (1.33-5.14)
	**Red eye**	258 (0.05)	729 (0.04)	1.36 (1.18-1.58)
	**Watery eyes**	5 (0.00)	33 (0.00)	0.66 (0.25-1.69)
**Reproductive health**	**Ejaculation difficulty**	14 (0.00)	33 (0.00)	1.55 (0.81-2.96)
	**Erectile dysfunction**	142 (0.03)	594 (0.03)	0.90 (0.75-1.09)
	**Menorrhagia**	280 (0.06)	1147 (0.06)	0.94 (0.82-1.07)
	**Menstrual changes**	323 (0.07)	1443 (0.07)	0.89 (0.79-1.00)
	**Premenstrual syndrome**	23 (0.00)	141 (0.01)	0.64 (0.41-1.01)
	**Vaginal discharge**	381 (0.08)	2027 (0.10)	0.77 (0.69-0.86)
	**Vaginal dryness**	18 (0.00)	74 (0.00)	1.03 (0.61-1.72)
	**Anorgasm**	0 (0.00)	2 (0.00)	0.39 (0.09-1.68)
	**Reduced libido**	10 (0.00)	46 (0.00)	0.87 (0.44-1.72)
**Other symptoms**	**Allergies**	284 (0.06)	1044 (0.05)	1.06 (0.92-1.21)
	**Angioedema**	63 (0.01)	207 (0.01)	1.20 (0.90-1.59)
	**Body ache**	203 (0.04)	277 (0.01)	2.64 (2.18-3.19)
	**Chills and shivering**	85 (0.02)	20 (0.00)	
	**Dizziness**	626 (0.13)	1628 (0.08)	1.48 (1.34-1.63)
	**Dry mouth**	60 (0.01)	110 (0.01)	2.14 (1.55-2.96)
	**Fever**	4795 (0.99)	508 (0.03)	30.22 (27.49-33.22)
	**Haemoptysis**	287 (0.06)	119 (0.01)	7.76 (6.21-9.69)
	**Hallucinations**	30 (0.01)	124 (0.01)	0.85 (0.55-1.31)
	**Headache**	9463 (1.95)	5799 (0.30)	5.65 (5.46-5.85)
	**Hot flushes**	43 (0.01)	311 (0.02)	0.55 (0.40-0.77)
	**Mouth ulcer**	104 (0.02)	222 (0.01)	1.86 (1.46-2.36)
	**Neurasthenia**	2 (0.00)	10 (0.00)	0.61 (0.12-3.21)
	**Polydipsia**	18 (0.00)	37 (0.00)	2.01 (1.12-3.60)
	**Polyuria**	391 (0.08)	1122 (0.06)	1.33 (1.18-1.49)
	**Seizures**	148 (0.03)	357 (0.02)	1.38 (1.12-1.69)
	**Sweating**	111 (0.02)	351 (0.02)	1.18 (0.95-1.48)
	**Swelling of lymph nodes**	124 (0.03)	327 (0.02)	1.33 (1.07-1.67)
	**Urinary incontinence**	267 (0.05)	875 (0.04)	1.14 (0.99-1.32)
	**Urinary retention**	148 (0.03)	162 (0.01)	2.28 (1.76-2.95)
	**Vertigo**	287 (0.06)	988 (0.05)	1.14 (1.00-1.31)

*Adjusted for age, sex, body mass index, smoking status, ethnicity, socioeconomic deprivation and symptom recorded at baseline between 3 and 12 months prior to index date

Supplementary Table 5: Recording of symptoms between four and twelve weeks after index date comparing between patients infected with SARS CoV-2 and propensity matched comparator cohort of patients no recorded evidence of SARS CoV-2 infection

Domain	Symptom	Cohort of patients infected with SARS CoV-2 (n=472,660) N (%)	Comparator cohort (n=1,888,003) N (%)	Adjusted HR (95% CI)*
Breathing	Orthopnoea	14 (0.00)	44 (0.00)	1.33 (0.73-2.44)
	Paroxysmal nocturnal dyspnoea	8 (0.00)	17 (0.00)	1.89 (0.81-4.39)
	Shortness of breath	2203 (0.47)	5633 (0.30)	1.64 (1.56-1.73)
	Shortness of breath at rest	76 (0.02)	139 (0.01)	2.26 (1.71-2.99)
	Shortness of breath on exertion	1881 (0.40)	5035 (0.27)	1.58 (1.50-1.67)
	Tachypnoea	16 (0.00)	22 (0.00)	2.94 (1.54-5.61)
	Wheezing	505 (0.11)	1398 (0.07)	1.45 (1.31-1.61)
Pain	Chest pain	2636 (0.56)	6092 (0.32)	1.73 (1.65-1.81)
	Neuropathic pain	142 (0.03)	565 (0.03)	1.00 (0.83-1.21)
	Pain	19413 (4.11)	64582 (3.42)	1.21 (1.19-1.23)
	Pleuritic chest pain	126 (0.03)	166 (0.01)	3.04 (2.41-3.83)
Circulation	Cold extremities	33 (0.01)	94 (0.00)	1.43 (0.96-2.12)
	Limb swelling	808 (0.17)	2420 (0.13)	1.41 (1.30-1.53)
	Orthostatic hypotension	126 (0.03)	322 (0.02)	1.62 (1.32-1.99)
	Palpitations	942 (0.20)	2282 (0.12)	1.65 (1.53-1.78)
	Presyncope	41 (0.01)	143 (0.01)	1.19 (0.84-1.68)
	Tachycardia	259 (0.05)	564 (0.03)	1.85 (1.60-2.15)
Fatigue	Fatigue	2264 (0.48)	4268 (0.23)	2.12 (2.02-2.23)
	Post exertional fatigue	0 (0.00)	1 (0.00)	
Cognitive health	Amnesia	255 (0.05)	762 (0.04)	1.36 (1.18-1.57)
	Brain fog	329 (0.07)	932 (0.05)	1.38 (1.21-1.56)
	Difficulty understanding	2 (0.00)	18 (0.00)	0.45 (0.10-1.94)
	Dysarthria	18 (0.00)	68 (0.00)	1.12 (0.66-1.88)
	Dysphasia	10 (0.00)	28 (0.00)	1.56 (0.76-3.22)
	Reading difficulty	0 (0.00)	0 (0.00)	
Movement	Apraxia	18 (0.00)	65 (0.00)	1.14 (0.68-1.93)
	Balance difficulty	67 (0.01)	228 (0.01)	1.20 (0.91-1.57)
	Tremors	129 (0.03)	448 (0.02)	1.18 (0.97-1.44)
Sleep	Excessive sleep	10 (0.00)	45 (0.00)	0.87 (0.44-1.74)
	Insomnia	1097 (0.23)	3168 (0.17)	1.37 (1.28-1.47)
Ear, nose and throat	Anosmia	192 (0.04)	128 (0.01)	5.96 (4.77-7.46)
	Cough	3363 (0.71)	7000 (0.37)	1.95 (1.88-2.04)
	Dysgeusia	106 (0.02)	96 (0.01)	4.36 (3.31-5.75)

	Dysphagia	355 (0.08)	976 (0.05)	1.46 (1.29-1.65)
	Ear pain	758 (0.16)	2765 (0.15)	1.09 (1.00-1.18)
	Hearing loss	6 (0.00)	20 (0.00)	1.14 (0.46-2.85)
	Hoarse voice	114 (0.02)	354 (0.02)	1.32 (1.07-1.63)
	Hyperacusis	3 (0.00)	16 (0.00)	0.68 (0.20-2.37)
	Nasal congestion	447 (0.09)	1437 (0.08)	1.24 (1.12-1.38)
	Phlegm	590 (0.12)	1424 (0.08)	1.71 (1.55-1.88)
	Sneezing	11 (0.00)	27 (0.00)	1.61 (0.80-3.25)
	Sore throat	771 (0.16)	3139 (0.17)	0.92 (0.85-1.00)
	Tinnitus	357 (0.08)	1176 (0.06)	1.22 (1.08-1.37)
Stomach and digestion	Abdominal pain	2882 (0.61)	10267 (0.54)	1.11 (1.07-1.16)
	Bloating	276 (0.06)	987 (0.05)	1.12 (0.98-1.28)
	Bowel incontinence	180 (0.04)	463 (0.02)	1.43 (1.20-1.70)
	Constipation	1059 (0.22)	3225 (0.17)	1.33 (1.24-1.43)
	Diarrhoea	801 (0.17)	2610 (0.14)	1.25 (1.15-1.35)
	Gastric reflux	975 (0.21)	3328 (0.18)	1.17 (1.09-1.25)
	gastritis	462 (0.10)	1309 (0.07)	1.40 (1.26-1.56)
	Nausea	346 (0.07)	1125 (0.06)	1.23 (1.09-1.39)
	Vomiting	404 (0.09)	1169 (0.06)	1.39 (1.24-1.55)
	Weight gain	82 (0.02)	357 (0.02)	0.91 (0.72-1.16)
	Weight loss	399 (0.08)	976 (0.05)	1.63 (1.45-1.83)
Muscles and joints	Asthenia	79 (0.02)	166 (0.01)	1.90 (1.46-2.49)
	Joint pain	755 (0.16)	2560 (0.14)	1.18 (1.09-1.28)
	Joint stiffness	11 (0.00)	43 (0.00)	1.02 (0.53-1.99)
	Muscle cramping	187 (0.04)	690 (0.04)	1.08 (0.92-1.27)
	Muscle pain	487 (0.10)	1385 (0.07)	1.40 (1.26-1.55)
	Muscle twitch	0 (0.00)	2 (0.00)	
	Paraesthesia	461 (0.10)	1488 (0.08)	1.24 (1.12-1.38)
Mental health	Anhedonia	344 (0.07)	923 (0.05)	1.45 (1.28-1.64)
	Anorexia	196 (0.04)	590 (0.03)	1.34 (1.14-1.57)
	Anxiety	5483 (1.16)	19550 (1.04)	1.10 (1.07-1.13)
	Anxiety and depression	2584 (0.55)	9962 (0.53)	1.02 (0.98-1.07)
	Depression	5042 (1.07)	19020 (1.01)	1.05 (1.02-1.08)
	Increased appetite	15 (0.00)	41 (0.00)	1.47 (0.82-2.67)
	loneliness	8 (0.00)	48 (0.00)	0.77 (0.36-1.63)
	Mood swings	72 (0.02)	264 (0.01)	1.08 (0.83-1.40)
	Post-traumatic stress disorder	38 (0.01)	188 (0.01)	0.80 (0.57-1.14)
Hair, skin and nails	Dry and scaly skin	199 (0.04)	535 (0.03)	1.50 (1.27-1.76)
	Hair loss	609 (0.13)	1066 (0.06)	2.26 (2.05-2.50)
	Hives	210 (0.04)	717 (0.04)	1.16 (0.99-1.35)
	Itchy skin	424 (0.09)	1280 (0.07)	1.34 (1.20-1.49)
	Nail changes	368 (0.08)	1118 (0.06)	1.31 (1.17-1.48)
	Purpura	16 (0.00)	61 (0.00)	1.10 (0.63-1.91)
	Rash	1717 (0.36)	5768 (0.31)	1.20 (1.14-1.26)
Eyes	Diplopia	65 (0.01)	249 (0.01)	1.05 (0.80-1.38)

	**Dry eye**	194 (0.04)	735 (0.04)	1.08 (0.92-1.26)
	**Eye pain**	53 (0.01)	163 (0.01)	1.28 (0.94-1.74)
	**Flashing lights and floaters**	45 (0.01)	146 (0.01)	1.26 (0.90-1.76)
	**Itchy eyes**	32 (0.01)	53 (0.00)	2.43 (1.56-3.77)
	**Photophobia**	12 (0.00)	39 (0.00)	1.21 (0.63-2.32)
	**Red eye**	392 (0.08)	1216 (0.06)	1.30 (1.16-1.46)
	**Watery eyes**	23 (0.00)	77 (0.00)	1.20 (0.75-1.92)
**Reproductive health**	**Ejaculation difficulty**	23 (0.00)	53 (0.00)	1.65 (1.01-2.69)
	**Erectile dysfunction**	381 (0.08)	1069 (0.06)	1.41 (1.26-1.59)
	**Menorrhagia**	593 (0.13)	2132 (0.11)	1.09 (0.99-1.19)
	**Menstrual changes**	732 (0.15)	2603 (0.14)	1.10 (1.01-1.19)
	**Premenstrual syndrome**	55 (0.01)	220 (0.01)	0.98 (0.73-1.31)
	**Vaginal discharge**	1254 (0.27)	3829 (0.20)	1.28 (1.20-1.37)
	**Vaginal dryness**	39 (0.01)	134 (0.01)	1.20 (0.84-1.72)
	**Anorgasm**	0 (0.00)	7 (0.00)	
	**Reduced libido**	46 (0.01)	107 (0.01)	1.69 (1.20-2.39)
**Other symptoms**	**Allergies**	670 (0.14)	2333 (0.12)	1.16 (1.06-1.26)
	**Angioedema**	116 (0.02)	369 (0.02)	1.26 (1.02-1.55)
	**Body ache**	200 (0.04)	516 (0.03)	1.52 (1.29-1.79)
	**Chills and shivering**	16 (0.00)	60 (0.00)	1.07 (0.62-1.86)
	**Dizziness**	840 (0.18)	2905 (0.15)	1.18 (1.09-1.27)
	**Dry mouth**	73 (0.02)	198 (0.01)	1.52 (1.16-1.98)
	**Fever**	497 (0.11)	944 (0.05)	2.12 (1.90-2.37)
	**Haemoptysis**	105 (0.02)	243 (0.01)	1.73 (1.38-2.17)
	**Hallucinations**	60 (0.01)	187 (0.01)	1.31 (0.98-1.75)
	**Headache**	3118 (0.66)	10031 (0.53)	1.23 (1.18-1.28)
	**Hot flushes**	150 (0.03)	500 (0.03)	1.21 (1.01-1.46)
	**Mouth ulcer**	141 (0.03)	393 (0.02)	1.43 (1.18-1.74)
	**Neurasthenia**	3 (0.00)	20 (0.00)	0.60 (0.18-2.01)
	**Polydipsia**	22 (0.00)	91 (0.00)	0.96 (0.61-1.54)
	**Polyuria**	632 (0.13)	2001 (0.11)	1.28 (1.17-1.40)
	**Seizures**	183 (0.04)	583 (0.03)	1.25 (1.06-1.48)
	**Sweating**	201 (0.04)	679 (0.04)	1.18 (1.01-1.38)
	**Swelling of lymph nodes**	192 (0.04)	586 (0.03)	1.28 (1.09-1.51)
	**Urinary incontinence**	567 (0.12)	1686 (0.09)	1.36 (1.24-1.49)
	**Urinary retention**	128 (0.03)	361 (0.02)	1.44 (1.17-1.76)
	**Vertigo**	482 (0.10)	1768 (0.09)	1.11 (1.00-1.23)

*Adjusted for age, sex, body mass index, smoking status, ethnicity, socioeconomic deprivation and symptom recorded at baseline between 3 and 12 months prior to index date

Supplementary Table 6: Comorbidity related risk factors associated with the development of Long COVID (WHO definition)

Risk factor	Sample size [N = 384,137]	Long COVID symptoms [N=29,869] (7.78) n (%)	Unadjusted HR (95% CI)	Adjusted HR* (95% CI)
**Comorbidities at baseline**				
**COPD**	8040	1741 (21.65)	2.71 (2.58-2.85)	1.55 (1.47-1.64)
**BPH**	4961	596 (12.01)	1.39 (1.28-1.51)	1.39 (1.28-1.52)
**Fibromyalgia**	4031	900 (22.33)	3.17 (2.97-3.39)	1.37 (1.28-1.47)
**Anxiety**	77753	10481 (13.48)	2.17 (2.12-2.23)	1.35 (1.31-1.39)
**Erectile Dysfunction**	16678	1551 (9.30)	1.15 (1.09-1.21)	1.33 (1.26-1.41)
**Depression**	83903	11222 (13.37)	2.22 (2.17-2.27)	1.31 (1.27-1.34)
**Migraine**	43043	5597 (13.00)	1.88 (1.83-1.94)	1.26 (1.22-1.30)
**Multiple Sclerosis**	791	98 (12.39)	1.52 (1.25-1.85)	1.26 (1.03-1.53)
**Coeliac Disease**	1669	207 (12.40)	1.58 (1.38-1.81)	1.25 (1.09-1.43)
**Learning Disability**	3283	295 (8.99)	1.22 (1.09-1.37)	1.24 (1.11-1.40)
**SLE**	485	68 (14.02)	1.94 (1.53-2.46)	1.24 (0.97-1.58)
**Chronic Pancreatitis**	276	50 (18.12)	2.07 (1.57-2.73)	1.23 (0.93-1.63)
**IBS**	27492	3691 (13.43)	1.84 (1.78-1.91)	1.20 (1.15-1.24)
**Endometriosis**	5727	800 (13.97)	1.92 (1.79-2.06)	1.19 (1.11-1.28)
**Low Hb**	20039	2683 (13.39)	1.78 (1.71-1.85)	1.18 (1.13-1.23)
**Ankylosing Spondylitis**	630	63 (10.00)	1.31 (1.03-1.68)	1.18 (0.92-1.51)
**Deafness**	3767	514 (13.64)	1.53 (1.40-1.67)	1.16 (1.06-1.27)
**Hepatitis C**	633	74 (11.69)	1.43 (1.14-1.80)	1.16 (0.92-1.46)
**Eating disorder**	3488	504 (14.45)	1.92 (1.75-2.09)	1.16 (1.06-1.27)
**Substance Misuse**	6449	775 (12.02)	1.69 (1.58-1.82)	1.15 (1.07-1.23)
**Back pain**	5483	718 (13.10)	1.76 (1.64-1.90)	1.15 (1.07-1.24)
**Asthma**	76946	8527 (11.08)	1.59 (1.55-1.63)	1.15 (1.12-1.18)
**Chronic Sinusitis**	6838	873 (12.77)	1.63 (1.52-1.74)	1.14 (1.07-1.22)
**PCOS**	9599	1166 (12.15)	1.73 (1.63-1.84)	1.14 (1.07-1.21)
**Coagulopathy**	4336	505 (11.65)	1.49 (1.36-1.63)	1.14 (1.04-1.25)
**Hemiplegia**	512	62 (12.11)	1.25 (0.97-1.60)	1.14 (0.88-1.46)
**Falls**	28946	3811 (13.17)	1.59 (1.53-1.64)	1.14 (1.09-1.18)
**Raynaud's**	4241	501 (11.81)	1.53 (1.40-1.67)	1.14 (1.03-1.27)
**Systemic Sclerosis**	116	15 (12.93)	1.75 (1.06-2.91)	1.14 (0.68-1.90)
**AIDS**	708	62 (8.76)	1.12 (0.87-1.43)	1.14 (0.89-1.47)
**IHD**	9837	1372 (13.95)	1.60 (1.52-1.69)	1.13 (1.06-1.21)
**Cardiomyopathy**	925	107 (11.57)	1.44 (1.20-1.75)	1.12 (0.92-1.36)
**Inflammatory Bowel Disease**	3799	414 (10.90)	1.37 (1.24-1.51)	1.12 (1.02-1.23)
**All Chronic Liver Disease**	10624	1322 (12.44)	1.65 (1.56-1.75)	1.12 (1.03-1.21)
**Diverticular Disease**	10610	1441 (13.58)	1.60 (1.52-1.69)	1.12 (1.06-1.19)

ADHD	1935	165 (8.53)	1.23 (1.05-1.43)	1.12 (0.96-1.30)
Polymyalgia Rheumatica	1499	237 (15.81)	1.79 (1.57-2.03)	1.12 (0.99-1.28)
Hay fever	70648	6879 (9.74)	1.36 (1.33-1.40)	1.11 (1.08-1.14)
Peptic Ulcer	4968	670 (13.49)	1.54 (1.43-1.67)	1.11 (1.02-1.20)
NAFLD	4837	632 (13.07)	1.80 (1.66-1.94)	1.11 (0.99-1.24)
Parkinson's	638	84 (13.17)	1.25 (1.01-1.54)	1.10 (0.89-1.37)
Unspecified Stroke	2576	384 (14.91)	1.49 (1.35-1.65)	1.09 (0.98-1.22)
Autoimmune Skin Conditions	5200	482 (9.27)	1.22 (1.11-1.33)	1.09 (0.99-1.19)
Cataract	11608	1571 (13.53)	1.47 (1.40-1.55)	1.09 (1.02-1.16)
VTE	7035	925 (13.15)	1.52 (1.42-1.62)	1.09 (1.02-1.17)
Cluster Headache	1359	166 (12.21)	1.63 (1.40-1.90)	1.09 (0.93-1.27)
Myocardial Infarction	4795	624 (13.01)	1.47 (1.35-1.59)	1.08 (0.99-1.18)
Chronic Fatigue Syndrome	1365	224 (16.41)	2.07 (1.82-2.36)	1.08 (0.95-1.24)
Osteoarthritis	41007	4848 (11.82)	1.47 (1.42-1.51)	1.08 (1.04-1.12)
Arrhythmia	24962	3151 (12.62)	1.42 (1.36-1.47)	1.07 (1.02-1.13)
TIA	3052	452 (14.81)	1.56 (1.42-1.71)	1.07 (0.97-1.18)
OSA	5538	628 (11.34)	1.47 (1.36-1.59)	1.07 (0.99-1.17)
Acne	53994	4841 (8.97)	1.18 (1.15-1.22)	1.06 (1.03-1.10)
Alcohol Misuse	19593	1925 (9.82)	1.24 (1.19-1.30)	1.06 (1.01-1.11)
AF	6557	880 (13.42)	1.38 (1.29-1.47)	1.05 (0.97-1.14)
AMD	2347	347 (14.78)	1.50 (1.35-1.66)	1.05 (0.94-1.17)
Epilepsy	5842	586 (10.03)	1.25 (1.15-1.35)	1.05 (0.96-1.14)
Valvular Heart Disease	4451	584 (13.12)	1.45 (1.33-1.57)	1.04 (0.96-1.14)
Eczema	75272	6942 (9.22)	1.23 (1.20-1.26)	1.04 (1.01-1.07)
Psoriasis	15764	1487 (9.43)	1.23 (1.17-1.30)	1.04 (0.99-1.10)
Hypertension	56002	5696 (10.17)	1.25 (1.22-1.29)	1.03 (0.99-1.06)
Congenital Heart Disease	2374	223 (9.39)	1.19 (1.05-1.36)	1.03 (0.90-1.17)
Aortic Aneurysm	657	86 (13.09)	1.36 (1.10-1.68)	1.03 (0.83-1.28)
Inflammatory Eye Disease	6528	636 (9.74)	1.19 (1.10-1.29)	1.02 (0.94-1.10)
Heart failure	2930	461 (15.73)	1.61 (1.47-1.77)	1.01 (0.92-1.12)
Alcoholic Liver disease	655	87 (13.28)	1.62 (1.31-2.00)	1.01 (0.80-1.26)
Haemorrhagic Stroke	985	109 (11.07)	1.17 (0.97-1.42)	1.00 (0.82-1.20)
Blindness	1512	202 (13.36)	1.40 (1.22-1.61)	1.00 (0.86-1.15)
Fragility Fracture	35823	3254 (9.08)	1.09 (1.05-1.13)	1.00 (0.96-1.04)
Gout	10838	949 (8.76)	1.02 (0.96-1.09)	1.00 (0.94-1.07)
Type 1 diabetes	2234	198 (8.86)	1.08 (0.94-1.25)	1.00 (0.87-1.16)
Cancer	15856	1671 (10.54)	1.21 (1.16-1.28)	0.99 (0.94-1.05)
Ischaemic Stroke	1624	218 (13.42)	1.34 (1.17-1.53)	0.99 (0.86-1.14)

**Other Pulmonary Disease**	1888	310 (16.42)	1.84 (1.64-2.06)	0.99 (0.88-1.11)
**Hypothyroidism**	16202	1822 (11.25)	1.42 (1.35-1.49)	0.99 (0.94-1.04)
**Type 2 diabetes**	24500	2547 (10.40)	1.26 (1.21-1.31)	0.99 (0.94-1.05)
**Glaucoma**	3694	402 (10.88)	1.20 (1.09-1.32)	0.98 (0.88-1.08)
**Hepatitis B**	1124	83 (7.38)	1.05 (0.84-1.30)	0.98 (0.79-1.22)
**Hyperthyroidism**	4039	435 (10.77)	1.35 (1.23-1.48)	0.98 (0.89-1.08)
**CKD**	9820	1288 (13.12)	1.34 (1.26-1.41)	0.97 (0.91-1.03)
**Serious Mental Illness**	3891	518 (13.31)	1.51 (1.39-1.65)	0.96 (0.88-1.05)
**PVD**	3426	418 (12.20)	1.47 (1.33-1.62)	0.94 (0.84-1.06)
**Sjogren's Syndrome**	433	60 (13.86)	1.71 (1.33-2.20)	0.94 (0.73-1.22)
**Rheumatoid Arthritis**	3180	379 (11.92)	1.39 (1.26-1.54)	0.92 (0.83-1.02)
**Diabetic Retinopathy**	9778	992 (10.15)	1.18 (1.11-1.25)	0.91 (0.84-0.98)
**Pernicious Anaemia**	974	137 (14.07)	1.63 (1.38-1.93)	0.91 (0.77-1.09)
**Vascular Dementia**	1148	148 (12.89)	1.00 (0.85-1.17)	0.91 (0.75-1.10)
**Alzheimer's disease**	2214	250 (11.29)	0.93 (0.82-1.06)	0.88 (0.74-1.04)
**Dementia Unspecified**	4901	574 (11.71)	0.98 (0.90-1.06)	0.76 (0.67-0.86)

*Adjusted hazard ratios estimated using a Cox proportional hazards model, adjusting for age, sex, ethnic group, socioeconomic status, index year, vaccination status, symptoms recorded before COVID-19, and comorbidities.

COPD=chronic obstructive pulmonary disease, BPH=benign prostatic hyperplasia, SLE=systemic lupus erythematosus, IBS = irritable bowel syndrome, AIDS= acquired immune deficiency syndrome, IHD=ischaemic heart disease, PCOS=poly cystic ovarian syndrome, NAFLD=non-alcoholic fatty liver disease, VTE = venous thromboembolism, ADHD= attention deficit hyperactivity disorder, TIA=transient ischaemic attack, OSA=obstructive sleep apnoea, AF=atrial fibrillation, AMD=age-related macular degeneration, CKD=chronic kidney disease, PVD=peripheral vascular disease

Supplementary Table 7: Risk factors associated with the development of Long COVID (definition based on a composite of significantly different symptoms between patients infected with SARS CoV-2 and propensity matched comparator cohort of patients no recorded evidence of SARS CoV-2 infection)

Risk factor	Sample size [N=384,137]	Long COVID symptoms [n=50,832] (7.78) n (%)	Unadjusted HR (95% CI)	Adjusted HR* (95% CI)
**Sex**				
Men	171593	16477 (9.60)	Ref	Ref
Women	212544	34355 (16.16)	1.72 (1.69-1.76)	1.47 (1.44-1.50)
**Age (years)**				
18-29	95969	10937 (11.40)	Ref	Ref
30 - 39	78302	9495 (12.13)	1.18 (1.15-1.21)	0.99 (0.96-1.02)
40 - 49	75349	9716 (12.89)	1.22 (1.19-1.25)	0.98 (0.95-1.01)
50 - 59	73262	9900 (13.51)	1.25 (1.22-1.28)	0.94 (0.91-0.97)
60 - 69	35932	5239 (14.58)	1.33 (1.29-1.37)	0.91 (0.87-0.94)
≥70	25323	5545 (21.90)	1.70 (1.65-1.76)	0.94 (0.89-0.99)
**Ethnicity**				
White	246717	34488 (13.98)	Ref	Ref
Asians	47788	6437 (13.47)	0.91 (0.84-0.98)	0.97 (0.90-1.05)
Black	15846	1883 (11.88)	1.05 (0.97-1.13)	1.23 (1.14-1.33)
Mixed	5976	695 (11.63)	1.04 (0.99-1.09)	1.10 (1.05-1.16)
Other	5438	711 (13.07)	0.98 (0.96-1.01)	1.09 (1.06-1.13)
Missing	62372	6618 (10.61)	0.74 (0.72-0.76)	0.91 (0.89-0.94)
**Body mass index (kg/m^2)**				
<18.5	10312	1260 (12.22)	0.91 (0.86-0.97)	0.94 (0.89-1.00)
18.5-25	117630	14878 (12.65)	Ref	Ref
25-30	109707	14837 (13.52)	1.09 (1.06-1.11)	1.07 (1.04-1.09)
>30	95799	15725 (16.41)	1.33 (1.30-1.36)	1.12 (1.10-1.15)
Missing	50689	4132 (8.15)	0.63 (0.61-0.65)	0.90 (0.87-0.94)
**Smoking status**				
Non-Smoker	141967	16786 (11.82)	Ref	Ref
Ex-Smoker	139294	21076 (15.13)	1.31 (1.28-1.34)	1.06 (1.04-1.09)
Current Smoker	85765	11742 (13.69)	1.26 (1.23-1.29)	1.08 (1.06-1.11)
Missing	17111	1228 (7.18)	0.59 (0.55-0.62)	0.89 (0.84-0.95)
**Socioeconomic status quintile (IMD)**				
1 (least deprived)	66564	7801 (11.72)	Ref	Ref
2	68657	8478 (12.35)	1.05 (1.02-1.08)	3.71 (3.60-3.82)
3	70699	9220 (13.04)	1.13 (1.10-1.16)	1.00 (1.00-1.00)
4	84002	11176 (13.30)	1.16 (1.13-1.20)	1.02 (0.99-1.05)
5 (most deprived)	87270	13045 (14.95)	1.25 (1.22-1.29)	1.05 (1.02-1.08)
Missing	6945	1112 (16.01)	1.30 (1.22-1.38)	1.05 (1.02-1.08)

**Symptoms recorded before COVID-19**	78880	19735 (25.02)	2.41 (2.37-2.45)	1.76 (1.73-1.79)
**Comorbidities**				
**Systemic Sclerosis**	116	26 (22.41)	2.05 (1.40-3.01)	1.47 (1.00-2.16)
**Anxiety**	77753	16684 (21.46)	2.01 (1.98-2.05)	1.38 (1.35-1.41)
**Erectile Dysfunction**	16678	2832 (16.98)	1.26 (1.21-1.31)	1.34 (1.28-1.40)
**Back pain**	5483	1334 (24.33)	2.00 (1.90-2.12)	1.31 (1.24-1.39)
**BPH**	4961	1031 (20.78)	1.47 (1.38-1.56)	1.30 (1.22-1.39)
**COPD**	8040	2380 (29.60)	2.22 (2.13-2.32)	1.25 (1.19-1.30)
**Learning Disability**	3283	509 (15.50)	1.27 (1.16-1.39)	1.24 (1.13-1.36)
**Ankylosing Spondylitis**	630	113 (17.94)	1.42 (1.18-1.71)	1.24 (1.03-1.49)
**Migraine**	43043	8714 (20.24)	1.71 (1.67-1.75)	1.21 (1.18-1.24)
**IBS**	27492	5924 (21.55)	1.76 (1.71-1.80)	1.19 (1.16-1.22)
**Fibromyalgia**	4031	1249 (30.98)	2.62 (2.47-2.77)	1.19 (1.12-1.26)
**Chronic Sinusitis**	6838	1462 (21.38)	1.64 (1.55-1.72)	1.18 (1.12-1.24)
**Endometriosis**	5727	1272 (22.21)	1.84 (1.74-1.94)	1.18 (1.12-1.25)
**Depression**	83903	17428 (20.77)	1.97 (1.93-2.00)	1.18 (1.15-1.21)
**Osteoarthritis**	41007	8832 (21.54)	1.66 (1.63-1.70)	1.18 (1.15-1.21)
**Hepatitis C**	633	125 (19.75)	1.48 (1.24-1.76)	1.17 (0.98-1.39)
**All Chronic Liver Disease**	10624	2248 (21.16)	1.70 (1.63-1.77)	1.17 (1.10-1.25)
**Falls**	28946	6556 (22.65)	1.69 (1.65-1.73)	1.17 (1.14-1.21)
**Coeliac Disease**	1669	323 (19.35)	1.45 (1.30-1.62)	1.16 (1.04-1.30)
**Autoimmune Skin Conditions**	5200	845 (16.25)	1.26 (1.18-1.35)	1.15 (1.07-1.23)
**Low Hb**	20039	4365 (21.78)	1.74 (1.68-1.79)	1.15 (1.11-1.18)
**Substance Misuse**	6449	1227 (19.03)	1.59 (1.51-1.69)	1.15 (1.08-1.22)
**Multiple Sclerosis**	791	154 (19.47)	1.43 (1.22-1.68)	1.15 (0.98-1.35)
**Hemiplegia**	512	108 (21.09)	1.37 (1.13-1.65)	1.15 (0.95-1.40)
**PCOS**	9599	1842 (19.19)	1.62 (1.54-1.69)	1.14 (1.09-1.20)
**ADHD**	1935	266 (13.75)	1.16 (1.03-1.30)	1.14 (1.01-1.28)
**Cluster Headache**	1359	277 (20.38)	1.62 (1.44-1.83)	1.14 (1.01-1.29)
**AIDS**	708	105 (14.83)	1.13 (0.93-1.37)	1.14 (0.94-1.38)
**VTE**	7035	1586 (22.54)	1.60 (1.52-1.68)	1.13 (1.07-1.19)
**Hay fever**	70648	11440 (16.19)	1.33 (1.30-1.36)	1.12 (1.10-1.15)
**Coagulopathy**	4336	830 (19.14)	1.47 (1.38-1.58)	1.12 (1.05-1.20)
**Eating disorder**	3488	737 (21.13)	1.67 (1.55-1.79)	1.12 (1.04-1.21)
**SLE**	485	105 (21.65)	1.77 (1.46-2.14)	1.12 (0.92-1.36)
**Chronic Pancreatitis**	276	73 (26.45)	1.84 (1.46-2.31)	1.11 (0.88-1.40)
**Arrhythmia**	24962	5490 (21.99)	1.53 (1.49-1.57)	1.10 (1.07-1.14)
**Haemorrhagic Stroke**	985	203 (20.61)	1.37 (1.19-1.57)	1.10 (0.96-1.26)
**Deafness**	3767	857 (22.75)	1.58 (1.47-1.69)	1.10 (1.02-1.18)
**Diverticular Disease**	10610	2452 (23.11)	1.67 (1.60-1.73)	1.10 (1.06-1.15)

Polymyalgia Rheumatica	1499	402 (26.82)	1.87 (1.69-2.06)	1.10 (0.99-1.21)
Asthma	76946	13430 (17.45)	1.44 (1.42-1.47)	1.10 (1.08-1.13)
Acne	53994	7902 (14.63)	1.12 (1.10-1.15)	1.09 (1.06-1.12)
Peptic Ulcer	4968	1114 (22.42)	1.57 (1.48-1.67)	1.09 (1.03-1.16)
Inflammatory Bowel Disease	3799	669 (17.61)	1.32 (1.22-1.43)	1.09 (1.01-1.18)
OSA	5538	1071 (19.34)	1.51 (1.43-1.61)	1.09 (1.02-1.15)
Blindness	1512	371 (24.54)	1.62 (1.46-1.79)	1.08 (0.97-1.20)
Inflammatory Eye Disease	6528	1162 (17.80)	1.31 (1.23-1.38)	1.08 (1.02-1.14)
Eczema	75272	11698 (15.54)	1.22 (1.20-1.25)	1.07 (1.04-1.09)
Alcohol Misuse	19593	3264 (16.66)	1.25 (1.20-1.29)	1.07 (1.03-1.11)
Raynaud's	4241	801 (18.89)	1.45 (1.35-1.55)	1.07 (0.98-1.17)
Valvular Heart Disease	4451	997 (22.40)	1.53 (1.44-1.63)	1.06 (1.00-1.14)
TIA	3052	759 (24.87)	1.65 (1.53-1.77)	1.06 (0.98-1.14)
Chronic Fatigue Syndrome	1365	333 (24.40)	1.83 (1.65-2.04)	1.06 (0.95-1.19)
IHD	9837	2245 (22.82)	1.60 (1.53-1.67)	1.05 (1.00-1.11)
Ischaemic Stroke	1624	398 (24.51)	1.56 (1.42-1.73)	1.05 (0.95-1.17)
Psoriasis	15764	2520 (15.99)	1.23 (1.19-1.28)	1.05 (1.00-1.09)
AMD	2347	603 (25.69)	1.64 (1.51-1.78)	1.05 (0.96-1.14)
Parkinson's disease	638	147 (23.04)	1.37 (1.17-1.61)	1.05 (0.89-1.23)
Gout	10838	1769 (16.32)	1.15 (1.09-1.20)	1.05 (1.00-1.10)
Cardiomyopathy	925	174 (18.81)	1.41 (1.21-1.63)	1.04 (0.89-1.21)
Cataract	11608	2712 (23.36)	1.59 (1.53-1.65)	1.04 (0.99-1.09)
Serious Mental Illness	3891	895 (23.00)	1.61 (1.50-1.72)	1.04 (0.98-1.12)
Epilepsy	5842	984 (16.84)	1.26 (1.19-1.35)	1.04 (0.98-1.11)
Type 2 diabetes	24500	4688 (19.13)	1.42 (1.38-1.46)	1.04 (1.00-1.08)
Hypertension	56002	10151 (18.13)	1.37 (1.34-1.40)	1.03 (1.00-1.06)
Hypothyroidism	16202	3204 (19.78)	1.51 (1.46-1.57)	1.03 (0.99-1.07)
Heart failure	2930	783 (26.72)	1.70 (1.58-1.82)	1.02 (0.95-1.11)
Myocardial Infarction	4795	1007 (21.00)	1.43 (1.35-1.52)	1.02 (0.95-1.10)
NAFLD	4837	1039 (21.48)	1.77 (1.67-1.89)	1.02 (0.94-1.11)
AF	6557	1523 (23.23)	1.48 (1.41-1.56)	1.01 (0.95-1.07)
PVD	3426	715 (20.87)	1.53 (1.42-1.64)	1.01 (0.92-1.10)
Unspecified Stroke	2576	629 (24.42)	1.52 (1.41-1.65)	1.01 (0.93-1.10)
Fragility Fracture	35823	5510 (15.38)	1.10 (1.07-1.14)	1.01 (0.98-1.04)
Cancer	15856	2943 (18.56)	1.30 (1.25-1.35)	1.00 (0.97-1.04)
Congenital Heart Disease	2374	363 (15.29)	1.14 (1.03-1.27)	1.00 (0.90-1.12)
Alcoholic Liver disease	655	153 (23.36)	1.70 (1.45-1.99)	1.00 (0.84-1.19)
Alzheimer's disease	2214	542 (24.48)	1.31 (1.21-1.43)	1.00 (0.89-1.12)
Hyperthyroidism	4039	753 (18.64)	1.39 (1.29-1.49)	1.00 (0.93-1.07)

**Glaucoma**	3694	739 (20.01)	1.37 (1.27-1.47)	0.99 (0.92-1.07)
**Vascular Dementia**	1148	308 (26.83)	1.35 (1.20-1.51)	0.99 (0.86-1.13)
**Chronic Kidney Disease**	9820	2319 (23.62)	1.51 (1.45-1.58)	0.99 (0.95-1.04)
**Other Pulmonary Disease**	1888	477 (25.26)	1.73 (1.58-1.90)	0.99 (0.90-1.09)
**Rheumatoid Arthritis**	3180	665 (20.91)	1.50 (1.39-1.62)	0.98 (0.90-1.05)
**Type 1 diabetes**	2234	341 (15.26)	1.12 (1.00-1.24)	0.98 (0.88-1.10)
**Diabetic Retinopathy**	9778	1893 (19.36)	1.38 (1.32-1.45)	0.97 (0.91-1.03)
**Aortic Aneurysm**	657	139 (21.16)	1.34 (1.13-1.58)	0.96 (0.81-1.13)
**Pernicious Anaemia**	974	236 (24.23)	1.70 (1.50-1.94)	0.95 (0.83-1.08)
**Hepatitis B**	1124	143 (12.72)	1.06 (0.90-1.25)	0.93 (0.79-1.10)
**Sjogren's Syndrome**	433	95 (21.94)	1.68 (1.37-2.05)	0.90 (0.73-1.10)
**Dementia Unspecified**	4901	1174 (23.95)	1.29 (1.21-1.36)	0.83 (0.76-0.92)

*Adjusted hazard ratios estimated using a Cox proportional hazards model, adjusting for age, sex, ethnic group, socioeconomic status, index year, vaccination status, symptoms recorded before COVID-19, and comorbidities.

IMD=Index of Multiple Deprivation, BPH=benign prostatic hyperplasia, COPD=chronic obstructive pulmonary disease, IBS = irritable bowel syndrome, PCOS=poly cystic ovarian syndrome, ADHD= attention deficit hyperactivity disorder, AIDS= acquired immune deficiency syndrome, VTE = venous thromboembolism, SLE=systemic lupus erythematosus, OSA=obstructive sleep apnoea, TIA=transient ischaemic attack, IHD=ischaemic heart disease, AMD=age-related macular degeneration, NAFLD=non-alcoholic fatty liver disease, AF=atrial fibrillation, PVD=peripheral vascular disease, CKD=chronic kidney disease

Supplementary Table 8: Consolidation of 115 symptoms into 50 symptoms to be included as indicators in latent class analysis

Consolidation of symptom codes were performed as follows
Step 1: Restriction to symptoms that were significantly associated with a history of SARS CoV-2 infection after 12 weeks of infection

Step 2: Consolidation of symptoms by combining or including symptoms:
(a) that are likely to fall under a single umbrella of symptom,
(b) that have mutually inclusive Snomed CT code lists,
(c) that are likely to be presented similarly and thereby potentially misclassified

Symptom	Consolidated Symptom
Shortness of breath	Shortness of breath (1)
Shortness of breath at rest	
Shortness of breath on exertion	
Tachypnoea	
Wheezing	Wheezing (2)
Chest pain	Chest pain (3)
Pleuritic chest pain	
Limb swelling	Limb swelling (4)
Palpitations	Palpitations / Tachycardia (5)
Tachycardia	
Presyncope	Presyncope / dizziness (6)
Dizziness	
Fatigue	Fatigue / asthenia (7)
Post exertional fatigue	
Asthenia	
Neurasthenia	
Amnesia	Cognitive problems (8)
Brain fog	
Difficulty understanding	
Reading difficulty	
Insomnia	Insomnia (9)
Anosmia	Anosmia (10)
Cough	Cough (11)
Dysphagia	Dysphagia (12)
Ear pain	Ear pain (13)
Hoarse voice	Hoarse voice (14)
Nasal congestion	Nasal congestion / Sneezing (15)
Sneezing	
Phlegm	Phlegm (16)
Abdominal pain	Abdominal pain (17)
Bloating	Bloating (18)
Bowel incontinence	Bowel incontinence (19)
Constipation	Constipation (20)

Diarrhoea	Diarrhoea (21)
Gastric reflux	Gastric reflux (22)
gastritis	
Nausea	Nausea / Vomiting (23)
Vomiting	
Weight loss	Weight loss (24)
Joint pain	Joint pain (25)
Paraesthesia	Paraesthesia (26)
Anorexia	Anorexia (27)
Anhedonia	Anxiety / depression (28)
Anxiety	
Anxiety and depression	
Depression	
Dry and scaly skin	Dry and scaly skin (29)
Hair loss	Hair loss (30)
Hives	Hives / Itchy skin (31)
Itchy skin	
Nail changes	Nail changes (32)
Purpura	Purpura / rash (33)
Rash	
Dry eye	Dry eye (34)
Red eye	Red/ watery eye (35)
Watery eyes	
Ejaculation difficulty	Sexual dysfunction (36)
Erectile dysfunction	
Anorgasm	
Reduced libido	
Menorrhagia	Menorrhagia (37)
Vaginal discharge	Vaginal discharge (38)
Allergies	Allergies / angioedema (39)
Angioedema	
Body ache	Body ache (40)
Chills and shivering	Chills and fever (41)
Fever	
Dry mouth	Dry mouth (42)
Haemoptysis	Haemoptysis (43)
Headache	Headache (44)
Hot flushes	Hot flushes (45)
Mouth ulcer	Mouth ulcer (46)
Polyuria	Polyuria (47)
Urinary incontinence	Urinary incontinence (48)
Urinary retention	Urinary retention (49)
Vertigo	Vertigo (0)

Supplementary Table 9A: Predicted latent class membership (model posterior probability)

Class	Dominant symptoms within the class	Predicted class membership
Class 1	Broad spectrum of symptoms including pain, fatigue and rash	80.0%
Class 2	Cough, shortness of breath and phlegm	5.8%
Class 3	Anxiety, depression and brain fog	14.2%

Supplementary Table 9B: item-response probabilities (ρ) of the symptom items conditional on latent class membership

	**Rho (ρ): item-response probabilities conditional on latent class membership**		
**Symptoms (Items)**	**Class 1**	**Class 2**	**Class 3**
Shortness of breath	0.0308	0.2881	0.0034
Wheezing	0.0033	0.1409	0.0000
Chest pain	0.0564	0.0351	0.0081
Limb swelling	0.0221	0.0197	0.0010
Palpitations	0.0268	0.0069	0.0057
Presyncope/dizziness	0.0247	0.0107	0.0036
Fatigue	0.0585	0.0318	0.0190
Cognitive problems	0.0038	0.0054	0.0443
Insomnia	0.0149	0.0065	0.0571
Anosmia	0.0044	0.0072	0.0007
Cough	0.0211	0.7103	0.0043
Dysphagia	0.0100	0.0074	0.0006
Ear pain	0.0196	0.0049	0.0040
Hoarse voice	0.0036	0.0055	0.0004
Nasal congestion/sneezing	0.0129	0.0152	0.0014
Phlegm	0.0001	0.1989	0.0000
Abdominal pain	0.0818	0.0145	0.0093
Bloating	0.0090	0.0013	0.0006
Bowel incontinence	0.0053	0.0013	0.0000
Constipation	0.0252	0.0110	0.0037
Diarrhoea	0.0222	0.0126	0.0045
Gastric reflux	0.0193	0.0168	0.0070
Nausea/vomiting	0.0179	0.0105	0.0030
Weight loss	0.0088	0.0034	0.0025
Joint pain	0.0227	0.0032	0.0039
Paraesthesia	0.0139	0.0047	0.0011
Anxiety/depression	0.0470	0.0459	1.0000

Anorexia	0.0040	0.0052	0.0066
Dry/scaly skin	0.0062	0.0000	0.0002
Hair loss	0.0303	0.0026	0.0039
Hives/itchy skin	0.0136	0.0099	0.0020
Nail changes	0.0105	0.0019	0.0006
Purpura/rash	0.0500	0.0154	0.0046
Dry eye	0.0065	0.0010	0.0004
Red/watery eye	0.0116	0.0014	0.0001
Sexual dysfunction	0.0118	0.0008	0.0022
Menorrhagia	0.0174	0.0027	0.0036
Vaginal discharge	0.0319	0.0077	0.0030
Allergies/angioedema	0.0282	0.0204	0.0033
Body ache	0.0055	0.0018	0.0007
Fever/Chills	0.0096	0.0231	0.0007
Dry mouth	0.0020	0.0018	0.0005
Haemoptysis	0.0015	0.0078	0.0003
Headache	0.0861	0.0465	0.0170
Hot flushes	0.0049	0.0008	0.0019
Mouth ulcer	0.0041	0.0009	0.0009
Polyuria	0.0166	0.0070	0.0015
Urinary incontinence	0.0150	0.0076	0.0016
Urinary retention	0.0035	0.0000	0.0004
Vertigo	0.0143	0.0022	0.0001

Supplementary Table 10: Baseline characteristics of patients with SARS CoV-2 infection, without persistent symptoms and with persistent symptoms and membership to three latent classes

		Latent Classes among those with persistent symptoms associated with SARS CoV-2 infection (n=50,832)		
	Patients without persistent symptoms	Class 1 (Broad spectrum of symptoms including pain, fatigue and rash)	Class 2 (Cough, shortness of breath and phlegm)	Class 3 (Anxiety, depression, insomnia & brain fog)
**Number of patients**	**(n=333,305)**	**(n=40,676) (80.0%)**	**(n=2,928) (5.8%)**	**(n=7,228) (14.2%)**
**Age at index (years)**				
**Mean (SD)**	43.1 (16.2)	47.3 (17.9)	53.5 (18.8)	38.2 (15.1)
**Median (IQR)**	41.0 (29.0 ; 54.0)	46.0 (33.0 ; 58.0)	53.0 (39.0 ; 65.0)	35.0 (25.0 ; 48.0)
**Categories, n (%)**				
**18-29**	85032 (25.5)	7932 (19.5)	378 (12.9)	2627 (36.3)
**30-39**	68807 (20.6)	7465 (18.3)	412 (14.1)	1618 (22.4)
**40-49**	65633 (19.7)	7874 (19.4)	497 (17.0)	1345 (18.6)
**50-59**	63362 (19.0)	8222 (20.2)	649 (22.2)	1029 (14.2)
**60-69**	30693 (9.2)	4447 (10.9)	413 (14.1)	379 (5.2)
**70+**	19778 (5.9)	4736 (11.6)	579 (19.8)	230 (3.2)
**Sex, n (%)**				
**Female**	178189 (53.46)	27446 (67.5)	1817 (62.1)	5092 (70.4)
**Male**	155116 (46.54)	13230 (32.5)	1111 (37.9)	2136 (29.6)
**Ethnicity, n (%)**				
**White**	212229 (63.7)	27138 (66.7)	2108 (72.0)	5242 (72.5)
**Asians**	41351 (12.4)	5574 (13.7)	347 (11.8)	516 (7.1)

**Black**	13963 (4.2)	1629 (4.0)	67 (2.3)	187 (2.6)
**Mixed**	5281 (1.6)	557 (1.4)	32 (1.1)	106 (1.5)
**Others**	4727 (1.4)	625 (1.5)	25 (0.8)	61 (0.8)
**Missing**	55754 (16.7)	5153 (12.7)	349 (11.9)	1116 (15.4)
**Socio-economic status IMD quintile, n (%)**				
**Quintile 1 (Least deprived)**	58763 (17.6)	6398 (15.7)	392 (13.4)	1011 (14.0)
**Quintile 2**	60179 (18.1)	6812 (16.7)	444 (15.2)	1222 (16.9)
**Quintile 3**	61479 (18.4)	7341 (18.1)	579 (19.8)	1300 (18.0)
**Quintile 4**	72826 (21.9)	8873 (21.8)	649 (22.2)	1654 (22.9)
**Quintile 5 (Most deprived)**	74225 (22.3)	10323 (25.4)	803 (27.4)	1919 (26.5)
**Missing**	5833 (1.7)	929 (2.3)	61 (2.0)	122 (1.7)
**Body mass index (kg/m2), n (%)**				
**<18.5**	9052 (2.7)	965 (2.4)	75 (2.6)	220 (3.0)
**18.5-24**	102752 (30.8)	11824 (29.1)	730 (24.9)	2324 (32.1)
**25-29**	94870 (28.5)	12114 (29.8)	881 (30.1)	1842 (25.5)
**30+**	80074 (24.0)	12639 (31.1)	1068 (36.5)	2018 (27.9)
**Missing**	46557 (14.0)	3134 (7.7)	174 (5.9)	824 (11.4)
**Smoking status**				
**Never smoked**	125181 (37.6)	13632 (33.5)	758 (25.9)	2396 (33.1)
**Ex-smoker**	118218 (35.5)	17076 (42.0)	1358 (46.4)	2642 (36.6)
**Current smoker**	74023 (22.2)	9060 (22.3)	769 (26.3)	1913 (26.5)
**Missing**	15883 (4.8)	908 (2.2)	43 (1.5)	277 (3.8)

Supplementary Table 11: Results of the multinomial logistic regression model for the polytomous class membership outcome

	Patients without persistent symptoms	Adjusted OR (95% CI) for each of the latent classes		
		Class 1 (Broad spectrum of symptoms including pain, fatigue and rash)	Class 2 (Cough, shortness of breath and phlegm)	Class 3 (Anxiety, depression, insomnia & brain fog)
**Number of patients**	**(n=333,305)**	**(n=40,676)**	**(n=2,928)**	**(n=7,228)**
**Age at index (years)**				
**18-29**		Ref	Ref	Ref
**30-39**		1.01 (0.97-1.04)	1.11 (0.96-1.28)	0.67 (0.63-0.72)
**40-49**		1.09 (1.05-1.13)	1.37 (1.19-1.57)	0.58 (0.54-0.63)
**50-59**		1.17 (1.13-1.22)	1.81 (1.58-2.07)	0.44 (0.41-0.48)
**60-69**		1.31 (1.26-1.37)	2.32 (2-2.69)	0.34 (0.3-0.38)
**70+**		2.16 (2.07-2.25)	5.12 (4.46-5.88)	0.3 (0.27-0.35)
**Sex**				
**Male**		Ref	Ref	Ref
**Female**		1.76 (1.72-1.8)	1.41 (1.3-1.52)	1.93 (1.83-2.04)
**Ethnicity**				
**White**		Ref	Ref	Ref
**Asians**		1.15 (1.12-1.19)	1 (0.89-1.13)	0.49 (0.45-0.54)
**Black**		0.9 (0.85-0.95)	0.48 (0.38-0.62)	0.48 (0.41-0.55)
**Mixed**		0.89 (0.82-0.98)	0.75 (0.53-1.07)	0.69 (0.57-0.84)
**Others**		1.15 (1.06-1.25)	0.65 (0.44-0.97)	0.49 (0.38-0.64)
**Missing**		0.89 (0.86-0.92)	0.87 (0.78-0.98)	0.83 (0.77-0.89)
**Socio-economic status IMD quintile**				
**Quintile 1 (Least deprived)**		Ref	Ref	Ref
**Quintile 2**	Ref	1.03 (0.99-1.06)	1.09 (0.95-1.25)	1.15 (1.06-1.25)

**Quintile 3**		1.07 (1.03-1.11)	1.41 (1.24-1.6)	1.2 (1.11-1.31)
**Quintile 4**		1.1 (1.06-1.13)	1.38 (1.21-1.56)	1.29 (1.19-1.39)
**Quintile 5 (Most deprived)**		1.24 (1.2-1.28)	1.68 (1.48-1.9)	1.45 (1.34-1.57)
**Missing**		1.42 (1.32-1.53)	1.6 (1.22-2.1)	1.18 (0.98-1.43)
**Body mass index (kg/m2)**				
**<18.5**		1 (0.93-1.07)	1.34 (1.05-1.7)	0.99 (0.86-1.14)
**18.5-24**		Ref	Ref	Ref
**25-29**		1.11 (1.08-1.15)	1.16 (1.05-1.29)	1.07 (1.01-1.14)
**30+**		1.31 (1.27-1.34)	1.59 (1.44-1.75)	1.29 (1.21-1.37)
**Missing**		0.82 (0.79-0.86)	0.84 (0.71-1.01)	0.88 (0.8-0.96)
**Smoking Status**				
**Never smoked**		Ref	Ref	Ref
**Ex-smoker**		1.18 (1.15-1.21)	1.43 (1.3-1.57)	1.25 (1.18-1.32)
**Current smoker**		1.11 (1.08-1.14)	1.56 (1.41-1.73)	1.35 (1.27-1.43)
**Missing**		0.8 (0.75-0.87)	0.83 (0.6-1.15)	0.89 (0.77-1.02)

Supplementary Table 12A: Snomed CT codes for confirmed COVID-19

DESCRIPTION	SNOMED CT CODE
Detection of 2019 novel coronavirus using polymerase chain reaction technique	1240511000000100
Detection of 2019-nCoV (novel coronavirus) using polymerase chain reaction technique	1240511000000100
Detection of SARS-CoV-2 (severe acute respiratory syndrome coronavirus 2) using polymerase chain reaction technique	1240511000000100
Detection of Wuhan 2019-nCoV (novel coronavirus) using polymerase chain reaction technique	1240511000000100
Wuhan 2019-nCoV (novel coronavirus) detected	1240581000000100
2019-nCoV (novel coronavirus) detected	1240581000000100
2019 novel coronavirus detected	1240581000000100
SARS-CoV-2 (severe acute respiratory syndrome coronavirus 2) detected	1240581000000100
SARS-CoV-2 (severe acute respiratory syndrome coronavirus 2) detection result positive	1240581000000100
SARS-CoV-2 (severe acute respiratory syndrome coronavirus 2) RNA (ribonucleic acid) detection result positive	1240581000000100
COVID-19 detected	1240581000000100
COVID-19 confirmed by laboratory test	1300721000000100
SARS-CoV-2 (severe acute respiratory syndrome coronavirus 2) RNA (ribonucleic acid) qualitative existence in specimen	1321301000000100
SARS-CoV-2 (severe acute respiratory syndrome coronavirus 2) antigen detection result positive	1322781000000100
2019-nCoV (novel coronavirus) antigen detection result positive	1322781000000100
SARS-CoV-2 (severe acute respiratory syndrome coronavirus 2) RNA (ribonucleic acid) detection result positive	1324601000000100
2019-nCoV (novel coronavirus) ribonucleic acid detected	1324601000000100
SARS-CoV-2 (severe acute respiratory syndrome coronavirus 2) RNA (ribonucleic acid) detection result positive at the limit of detection	1324881000000100
2019-nCoV (novel coronavirus) detection result positive at the limit of detection	1324881000000100
Detection of SARS-CoV-2 (severe acute respiratory syndrome coronavirus 2) antigen	871553007
Detection of ribonucleic acid of 2019 novel coronavirus in nasopharyngeal swab	871556004
Detection of RNA (ribonucleic acid) of SARS-CoV-2 (severe acute respiratory syndrome coronavirus 2) in nasopharyngeal swab	871556004
Detection of RNA (ribonucleic acid) of SARS-CoV-2 (severe acute respiratory syndrome coronavirus 2) in oropharyngeal swab	871557008
Detection of RNA (ribonucleic acid) of SARS-CoV-2 (severe acute respiratory syndrome coronavirus 2) using polymerase chain reaction	871560001
Detection of ribonucleic acid of COVID-19 using polymerase chain reaction	871560001

Supplementary Table 12B: Snomed CT codes for suspected COVID-19

DESCRIPTION	SNOMED CT CODE
Suspected disease caused by Wuhan 2019-nCoV (novel coronavirus)	1240761000000100
Myocarditis caused by 2019 novel coronavirus	1240531000000100
Disease caused by 2019 novel coronavirus	1240751000000100
Otitis media caused by Wuhan 2019-nCoV (novel coronavirus)	1240521000000100
Upper respiratory tract infection caused by 2019-nCoV (novel coronavirus)	1240541000000100
Otitis media caused by 2019 novel coronavirus	1240521000000100
Disease caused by Wuhan 2019-nCoV (novel coronavirus)	1240751000000100
Pneumonia caused by 2019 novel coronavirus	1240551000000100
Telephone consultation for suspected 2019-nCoV (novel coronavirus)	1240451000000100
Gastroenteritis caused by Wuhan 2019-nCoV (novel coronavirus)	1240571000000100
Telephone consultation for suspected Wuhan 2019-nCoV (novel coronavirus)	1240451000000100
Upper respiratory tract infection caused by Wuhan 2019-nCoV (novel coronavirus)	1240541000000100
Encephalopathy caused by Wuhan 2019-nCoV (novel coronavirus)	1240561000000100
Myocarditis caused by 2019-nCoV (novel coronavirus)	1240531000000100
Pneumonia caused by Wuhan 2019-nCoV (novel coronavirus)	1240551000000100
Encephalopathy caused by 2019-nCoV (novel coronavirus)	1240561000000100
Confirmed 2019-nCoV (novel coronavirus) infection	12802201000006100
Pneumonia caused by SARS-CoV-2 (severe acute respiratory syndrome coronavirus 2)	1240551000000100
Gastroenteritis caused by SARS-CoV-2 (severe acute respiratory syndrome coronavirus 2)	1240571000000100
Possible COVID-19	1240761000000100
Probable COVID-19 confirmed using clinical diagnostic criteria	1300731000000100
Myocarditis caused by SARS-CoV-2 (severe acute respiratory syndrome coronavirus 2)	1240531000000100
Suspected COVID-19 caused by SARS-CoV-2 (severe acute respiratory syndrome coronavirus 2)	1240761000000100
Telephone consultation for suspected SARS-CoV-2 (severe acute respiratory syndrome coronavirus 2)	1240451000000100
COVID-19 confirmed clinically	1300731000000100
Otitis media caused by SARS-CoV-2 (severe acute respiratory syndrome coronavirus 2)	1240521000000100
Upper respiratory tract infection caused by SARS-CoV-2 (severe acute respiratory syndrome coronavirus 2)	1240541000000100
Telephone consultation for suspected 2019 novel coronavirus	1240451000000100
Disease caused by 2019-nCoV (novel coronavirus)	1240751000000100
COVID-19 severity score	1300631000000100
COVID-19 confirmed using clinical diagnostic criteria	1300731000000100
Myocarditis caused by Wuhan 2019-nCoV (novel coronavirus)	1240531000000100
Suspected disease caused by 2019-nCoV (novel coronavirus)	1240761000000100
Infection of upper respiratory tract caused by 2019 novel coronavirus	1240541000000100
Gastroenteritis caused by 2019 novel coronavirus	1240571000000100
Suspected COVID-19	1240761000000100
Signposting to CHMS (COVID-19 Home Management Service)	1321231000000100

COVID-19	1240751000000100
Encephalopathy caused by SARS-CoV-2 (severe acute respiratory syndrome coronavirus 2)	1240561000000100
SARS-CoV infection	398447004
Otitis media caused by 2019-nCoV (novel coronavirus)	1240521000000100
Gastroenteritis caused by 2019-nCoV (novel coronavirus)	1240571000000100
Suspected disease caused by 2019 novel coronavirus	1240761000000100
Pneumonia caused by 2019-nCoV (novel coronavirus)	1240551000000100
Encephalopathy caused by 2019 novel coronavirus	1240561000000100
Suspected 2019-nCoV (novel coronavirus) infection	12802181000006100
COVID-19 caused by SARS-CoV-2 (severe acute respiratory syndrome coronavirus 2)	1240751000000100
2019-nCoV (novel coronavirus) IgG detected	1321541000000100
2019-nCoV (novel coronavirus) IgA detected	1321761000000100
2019-nCoV (novel coronavirus) antibody detection result positive	1322871000000100
SARS-CoV-2 (severe acute respiratory syndrome coronavirus 2) antibody detection result positive	1322871000000100
SARS-CoV-2 (severe acute respiratory syndrome coronavirus 2) IgA detection result positive	1321761000000100
Serotype SARS-CoV-2 (severe acute respiratory syndrome coronavirus 2)	1240421000000100
Pneumonia caused by SARS-CoV-2 (severe acute respiratory syndrome coronavirus 2)	882784691000119000
Disease caused by 2019-nCoV	840539006
Pneumonia caused by 2019 novel coronavirus	882784691000119000
Infection of upper respiratory tract caused by 2019 novel coronavirus	1240541000000100
Encephalopathy due to COVID-19	1240561000000100
Myocarditis due to disease caused by SARS-CoV-2 (severe acute respiratory syndrome coronavirus 2)	1240531000000100
Otitis media due to COVID-19	1240521000000100
Encephalopathy due to disease caused by SARS-CoV-2 (severe acute respiratory syndrome coronavirus 2)	1240561000000100
Detection of SARS-CoV-2 (severe acute respiratory syndrome coronavirus 2)	871562009
Lymphopenia due to COVID-19	866151004
Suspected COVID-19	840544004
COVID-19 lower respiratory infection	880529761000119000
Fever caused by SARS-CoV-2 (severe acute respiratory syndrome coronavirus 2)	119751000146104
Lymphocytopenia due to SARS-CoV-2 (severe acute respiratory syndrome coronavirus 2)	866151004
Acute respiratory distress syndrome due to disease caused by SARS-CoV-2 (severe acute respiratory syndrome coronavirus 2)	674814021000119000
Detection of SARS-CoV-2 (severe acute respiratory syndrome coronavirus 2) antibody	871552002
SARS-CoV-2 (severe acute respiratory syndrome coronavirus 2) IgG detection result positive	1321541000000100
Myocarditis due to COVID-19	1240531000000100
Pneumonia caused by 2019-nCoV (novel coronavirus)	882784691000119000
Disease caused by 2019 novel coronavirus	840539006
COVID-19 pneumonia	882784691000119000

Dyspnoea caused by SARS-CoV-2 (severe acute respiratory syndrome coronavirus 2)	119981000146107
Detection of COVID-19	871562009
Signposting to Your COVID Recovery	1325021000000100
Serotype 2019-nCoV (novel coronavirus)	1240421000000100
Suspected disease caused by 2019-nCoV (novel coronavirus)	840544004
Suspected disease caused by 2019 novel coronavirus	840544004
Sepsis due to disease caused by COVID-19	870588003
COVID-19 acute bronchitis	138389411000119000
Detection of 2019 novel coronavirus antibody	871552002
COVID-19	840539006
Acute COVID-19 infection	1325171000000100
Sepsis due to disease caused by SARS-CoV-2 (severe acute respiratory syndrome coronavirus 2)	870588003
Lower respiratory infection caused by SARS-CoV-2 (severe acute respiratory syndrome coronavirus 2)	880529761000119000
COVID-19 acute respiratory distress syndrome	674814021000119000
Ongoing symptomatic COVID-19	1325181000000100
Asymptomatic SARS-CoV-2 (severe acute respiratory syndrome coronavirus 2) infection	189486241000119000
Suspected disease caused by SARS-CoV-2 (severe acute respiratory syndrome coronavirus 2)	840544004
SARS-CoV-2 (severe acute respiratory syndrome coronavirus 2) IgG qualitative existence in specimen	1321321000000100
SARS-CoV-2 (severe acute respiratory syndrome coronavirus 2) IgM detection result positive	1321551000000100
Cardiomyopathy due to disease caused by SARS-CoV-2 (severe acute respiratory syndrome coronavirus 2)	119731000146105
Acute hypoxemic respiratory failure due to disease caused by SARS-CoV-2 (severe acute respiratory syndrome coronavirus 2)	870590002
Asymptomatic COVID-19	189486241000119000
Anti-SARS-CoV-2 IgG	870361009
Antibody to SARS-CoV-2 (severe acute respiratory syndrome coronavirus 2)	1240401000000100
Otitis media due to disease caused by SARS-CoV-2 (severe acute respiratory syndrome coronavirus 2)	1240521000000100
Post-COVID-19 syndrome	1325161000000100
Acute kidney injury due to disease caused by SARS-CoV-2 (severe acute respiratory syndrome coronavirus 2)	870589006
Acute bronchitis caused by SARS-CoV-2 (severe acute respiratory syndrome coronavirus 2)	138389411000119000
Possible COVID-19	840544004

Supplementary Table 13: List of symptoms outcomes considered

Domain	Symptom	Component of the primary outcome: WHO case definition for Long COVID	Component of the secondary outcome: significantly reported higher among patients with COVID-19
Breathing	Orthopnoea		
	Paroxysmal nocturnal dyspnoea		
	Shortness of breath	X	X
	Shortness of breath at rest		X
	Shortness of breath on exertion		X
	Tachypnoea		
	Wheezing		X
Pain	Chest pain	X	X
	Neuropathic pain		
	Pain		X
	Pleuritic chest pain		X
Circulation	Cold extremities		
	Limb swelling		X
	Orthostatic hypotension		
	Palpitations	X	X
	Presyncope		
	Tachycardia		X
Fatigue	Fatigue	X	X
	Post exertional fatigue	X	
Cognitive health	Amnesia	X	
	Brain fog	X	X
	Difficulty understanding		
	Dysarthria		
	Dysphasia		
	Reading difficulty		
Movement	Apraxia		
	Balance difficulty		
	Tremors		
Sleep	Excessive sleep	X	
	Insomnia	X	X
Ear, nose and throat	Anosmia	X	X
	Cough	X	X
	Dysgeusia	X	
	Dysphagia		X
	Ear pain		X
	Hearing loss	X	
	Hoarse voice		X
	Hyperacusis	X	
	Nasal congestion		X
	Phlegm		X

	Sneezing		X
	Sore throat		
	Tinnitus		
Stomach and digestion	Abdominal pain	X	X
	Bloating		X
	Bowel incontinence		X
	Constipation	X	X
	Diarrhoea	X	X
	Gastric reflux	X	
	gastritis	X	X
	Nausea		X
	Vomiting		X
	Weight gain		
	Weight loss		X
Muscles and joints	Asthenia		X
	Joint pain	X	X
	Joint stiffness		
	Muscle cramping	X	
	Muscle pain	X	
	Muscle twitch		
	Paraesthesia	X	X
Mental health	Anhedonia		X
	Anorexia		X
	Anxiety		X
	Anxiety and depression	X	
	Depression	X	X
	Increased appetite		
	loneliness		
	Mood swings		
	Post-traumatic stress disorder		
Hair, skin and nails	Dry and scaly skin		X
	Hair loss		X
	Hives		
	Itchy skin		X
	Nail changes		X
	Purpura		
	Rash		X
Eyes	Diplopia	X	
	Dry eye		X
	Eye pain		
	Flashing lights and floaters		
	Itchy eyes		
	Photophobia		
	Red eye		X
	Watery eyes		
	Ejaculation difficulty		X

Reproductive health	Erectile dysfunction		X
	Menorrhagia	X	X
	Menstrual changes	X	
	Premenstrual syndrome	X	
	Vaginal discharge		X
	Vaginal dryness		
	Anorgasm		
	Reduced libido		X
Other symptoms	Allergies	X	X
	Angioedema		
	Body ache		X
	Chills and shivering		
	Dizziness	X	X
	Dry mouth		X
	Fever	X	X
	Haemoptysis		X
	Hallucinations		
	Headache	X	X
	Hot flushes		X
	Mouth ulcer		X
	Neurasthenia		
	Polydipsia		
	Polyuria		X
	Seizures		
	Sweating		
	Swelling of lymph nodes		
	Urinary incontinence		X
	Urinary retention		X
	Vertigo		X
