# Supplementary material for: Symptoms and risk factors for long COVID in non-hospitalized adults
Source: Nat Med. 2022 Jul 25;28(8):1706–14. doi: 10.1038/s41591-022-01909-w (PMC9388369; doi:10.1038/s41591-022-01909-w)
Supplement: Supplementary file 2 — Reporting Summary [file 41591_2022_1909_MOESM2_ESM.pdf]

nature research

Corresponding author(s): Prof Krishnarajah Nirantharakumar

Last updated by author(s): Jun 13, 2022

Reporting Summary

Nature Research wishes to improve the reproducibility of the work that we publish. This form provides structure for consistency and transparency in reporting. For further information on Nature Research policies, see our Editorial Policies and the Editorial Policy Checklist.

Statistics

For all statistical analyses, confirm that the following items are present in the figure legend, table legend, main text, or Methods section.

n/a	Confirmed	
☐	☒	The exact sample size (*n*) for each experimental group/condition, given as a discrete number and unit of measurement
☒	☐	A statement on whether measurements were taken from distinct samples or whether the same sample was measured repeatedly
☐	☒	The statistical test(s) used AND whether they are one- or two-sided<br>*Only common tests should be described solely by name; describe more complex techniques in the Methods section.*
☐	☒	A description of all covariates tested
☐	☒	A description of any assumptions or corrections, such as tests of normality and adjustment for multiple comparisons
☐	☒	A full description of the statistical parameters including central tendency (e.g. means) or other basic estimates (e.g. regression coefficient) AND variation (e.g. standard deviation) or associated estimates of uncertainty (e.g. confidence intervals)
☐	☒	For null hypothesis testing, the test statistic (e.g. *F*, *t*, *r*) with confidence intervals, effect sizes, degrees of freedom and *P* value noted<br>*Give P values as exact values whenever suitable.*
☒	☐	For Bayesian analysis, information on the choice of priors and Markov chain Monte Carlo settings
☒	☐	For hierarchical and complex designs, identification of the appropriate level for tests and full reporting of outcomes
☒	☐	Estimates of effect sizes (e.g. Cohen's *d*, Pearson's *r*), indicating how they were calculated

Our web collection on statistics for biologists contains articles on many of the points above.

Software and code

Policy information about availability of computer code

Data collection	Data extraction for epidemiological research (DExtER) tool version 2021.09 was used for data extraction
Data analysis	Stata IC version 16 and R version 4.0.4 were used for data analysis.<br>Stata and R codes are available at https://github.com/AnuSub/Stata-and-R-codes.

For manuscripts utilizing custom algorithms or software that are central to the research but not yet described in published literature, software must be made available to editors and reviewers. We strongly encourage code deposition in a community repository (e.g. GitHub). See the Nature Research guidelines for submitting code & software for further information.

Data

Policy information about availability of data

All manuscripts must include a data availability statement. This statement should provide the following information, where applicable:

- Accession codes, unique identifiers, or web links for publicly available datasets
- A list of figures that have associated raw data
- A description of any restrictions on data availability

Access to anonymised patient data from CPRD is subject to a data sharing agreement (DSA) containing detailed terms and conditions of use following protocol approval from MHRA Independent Scientific Advisory Committee (ISAC). This study specific analysable data set is therefore not publicly available and can be requested from the corresponding author at K.Nirantharan@bham.ac.uk subject to Research Data Governance (RDG) approvals. Details about ISAC applications and data costs are available on the CPRD website (cprd.com).

Field-specific reporting

Please select the one below that is the best fit for your research. If you are not sure, read the appropriate sections before making your selection.

☒ Life sciences ☐ Behavioural & social sciences ☐ Ecological, evolutionary & environmental sciences

For a reference copy of the document with all sections, see nature.com/documents/nr-reporting-summary-flat.pdf

Life sciences study design

All studies must disclose on these points even when the disclosure is negative.

Sample size	A total of 486,149 non-hospitalised individuals had a coded record of confirmed SARS CoV-2 infection, and 8,030,224 had no recorded evidence of suspected or confirmed SARS CoV-2 infection in the study period. From the pool of patients with no recorded evidence of infection, 1,944,580 individuals were propensity score matched to patients with a record of infection. Of these patients, 384,137 and 1,501,689 patients with and without a record of SARS CoV-2 infection had a minimum follow-up of 12 months. This resulted in over 90% statistical power to detect the observed difference in the hazard of composite symptom outcome (aHR: 1.26, 1.25-1.28)
Data exclusions	(1) As per prespecified protocol, patients aged 18 years or below on index date were excluded from the study since our study's aim was to explore longer term symptoms presented by adult patients with SARS CoV-2 infection<br>(2) As per prespecified protocol, patients who were registered with a general practice for less than 12 months were excluded from the study since they may not have sufficient time window to record symptoms, co-morbidities and other risk factors at baseline.<br>(3) As per prespecified protocol, practices contributing data that were not of research quality were excluded from the study<br>(4) As per prespecified protocol. patients with a record of hospitalisation 14 days before or 42 days after infection (within 28 days of infection with a ±14 day grace period for clinical coding delays) were excluded from the study, since our study focussed on non-hospitalised adults.<br>(5) As per prespecified protocol, patients without a minimum follow-up period of four and twelve weeks were excluded from the symptom outcome analysis during the four to twelve weeks period after infection (period of "ongoing symptomatic COVID-19") and twelve weeks after infection (period of "post-COVID-19 conditions or "long COVID"), respectively so that patients were eligible for follow-up at the start of the time window studied.
Replication	Data for this study was extracted in an analysable format from CPRD AURUM database using the in-house DExtER software, which provides a non-invasive solution to generate quality datasets through a process that can be verified and reproducible. Stata and R codes are available at https://github.com/AnuSub/Stata-and-R-codes.
Randomization	This was not a randomised controlled study. However, in this retrospective cohort study, we propensity score matched patients with and without a record of SARS CoV-2 infection using a logistic regression model with a caliper of width 0.2, including a comprehensive range of covariates such as age, sex, body mass index, smoking status, socioeconomic deprivation, ethnicity, symptoms and comorbidities recorded at baseline. The result of this was well balanced cohorts in terms of baseline covariates, evidence by the kernel density plots of propensity scores for the two cohorts before and after matching, and the estimated standardised mean differences for each of the baseline covariates, which were all < 0.1.
Blinding	Blinding was not applicable in this study as the data for exposure and outcome were both collected retrospectively from primary care records. Investigators were unable to be blinded to patients' exposure status at the analysis stage since propensity score matching to match baseline covariates between the two groups required the investigators to know the exposure status of each patient.

Reporting for specific materials, systems and methods

We require information from authors about some types of materials, experimental systems and methods used in many studies. Here, indicate whether each material, system or method listed is relevant to your study. If you are not sure if a list item applies to your research, read the appropriate section before selecting a response.

Materials & experimental systems

n/a	Involved in the study	
☒	☐	Antibodies
☒	☐	Eukaryotic cell lines
☒	☐	Palaeontology and archaeology
☒	☐	Animals and other organisms
☐	☒	Human research participants
☐	☒	Clinical data
☒	☐	Dual use research of concern

Methods

n/a	Involved in the study	
☒	☐	ChIP-seq
☒	☐	Flow cytometry
☒	☐	MRI-based neuroimaging

Human research participants

Policy information about studies involving human research participants

Population characteristics	The mean age of all eligible patients included in our study was 43.8 years (SD 16.9) and 55.3% were female. 64.7% were

Population characteristics	white, 12.2% were Asian and 4.0% were black, and ethnicity data were missing for 16.2%. 53.8% were overweight or obese (with BMI data missing for 13.0%) and 22.5% were current smokers (with smoking data missing for 4.3%). The most common comorbidities were depression (22.1%), anxiety (20.3%), asthma (20.1%), eczema (19.5%), and hay fever (18.1%).
Recruitment	We conducted a population-based retrospective matched cohort study data from the Medicines and Healthcare products Regulatory Agency (MHRA) Clinical Practice Research Datalink (CPRD) Aurum. CPRD Aurum is an anonymised database of primary care medical records of over seven million actively registered patients in general practices that use the EMIS clinical information system . A total of 486,149 non-hospitalised individuals had a coded record of confirmed SARS CoV-2 infection, and 8,030,244 had no recorded evidence of either suspected or confirmed SARS CoV-2 infection during the study period, which formed the pool of control patients. A limitation of our study is potential misclassification bias due to retrospective recruitment of patients with and without a record of infection based on Snomed CT codes. Community testing for SARS CoV-2 was limited during the first surge of the pandemic in the UK, and many non-hospitalised individuals with COVID-19 were not tested. It is therefore possible that some members of our comparator cohort had been infected with SARS CoV-2 but had simply not been tested. We attempted to account for this bias by excluding individuals from the comparator cohort if they had a coded diagnosis of suspected COVID-19. However, this is unlikely to be 100% sensitive in identifying individuals with unverified COVID-19 from the comparator cohort, which would potentially have the effect of attenuating the observed effect sizes.
Ethics oversight	CPRD obtains annual research ethics approval from the UK's Health Research Authority (HRA) Research Ethics Committee (REC) (East Midlands – Derby, REC reference number 05/MRE04/87) to receive and supply patient data for public health research. Therefore, no additional ethics approval is required for observational studies using CPRD Aurum data for public health research, subject to individual research protocols meeting CPRD data governance requirements. The use of CPRD Aurum data for the study has been approved by the CPRD Independent Scientific Advisory Committee (reference: 21_000423).

Note that full information on the approval of the study protocol must also be provided in the manuscript.

Clinical data

Policy information about clinical studies

All manuscripts should comply with the ICMJE guidelines for publication of clinical research and a completed CONSORT checklist must be included with all submissions.

Clinical trial registration	This is not a clinical trial.
Study protocol	http://dx.doi.org/10.1136/bmjopen-2021-060413
Data collection	We conducted a population-based retrospective matched cohort study between 31st January 2020 and 15th April 2021 using data from the Medicines and Healthcare products Regulatory Agency (MHRA) Clinical Practice Research Datalink (CPRD) Aurum. CPRD Aurum is an anonymised database of primary care medical records of over seven million actively registered patients in general practices that use the EMIS clinical information system. It captures data on patient demographics, diagnoses, symptoms, prescriptions, referrals, and tests. Structures data on diagnoses, symptoms, and referrals are recorded using Snomed CT coding terminology.
Outcomes	We identified 115 relevant symptoms coded within primary care records through a systematic review and meta-analysis of long COVID symptoms, a scoping search of long COVID clinical assessment questionnaires, qualitative interviews with patients, a clinician survey, and refinement of the symptom list using psychometric methods. These were groups into 15 domains: (1) breathing, (2) pain, (3) circulation, (4) fatigue, (5) cognitive health, (6) movement, (7) sleep, (8) ear, nose, and throat, (9) stomach and digestion, (10) muscles and joints, (11) mental health, (12) hair, skin and nails, (13) eyes, (14) reproductive health, and (15) other symptoms. Our primary outcome definition of long COVID was predefined as the presence of at least one symptom included in the WHO case definition at ≥ 12 weeks post-infection, Our secondary outcome definition of long COVID was derived post-hoc as the presence of at least one symptom that was statistically associated with SARS CoV-2 infection at ≥ 12 weeks post-infection within this study.
